# Brain-wide reconstruction of inhibitory circuits after traumatic brain injury

**DOI:** 10.1038/s41467-022-31072-2

**Published:** 2022-06-14

**Authors:** Jan C. Frankowski, Alexa Tierno, Shreya Pavani, Quincy Cao, David C. Lyon, Robert F. Hunt

**Affiliations:** 1 Department of Anatomy & Neurobiology, University of California, Irvine, CA 92697 USA; 2 Epilepsy Research Center, University of California, Irvine, CA 92697 USA; 3 Sue and Bill Gross Stem Cell Research Center, University of California, Irvine, CA 92697 USA; 4 Center for the Neurobiology of Learning and Memory, University of California, Irvine, Irvine, CA 92697 USA; 5 Center for Neural Circuit Mapping, University of California, Irvine, Irvine, CA 92697 USA

**Keywords:** Epilepsy, Neural circuits, Regeneration and repair in the nervous system

## Abstract

Despite the fundamental importance of understanding the brain’s wiring diagram, our knowledge of how neuronal connectivity is rewired by traumatic brain injury remains remarkably incomplete. Here we use cellular resolution whole-brain imaging to generate brain-wide maps of the input to inhibitory neurons in a mouse model of traumatic brain injury. We find that somatostatin interneurons are converted into hyperconnected hubs in multiple brain regions, with rich local network connections but diminished long-range inputs, even at areas not directly damaged. The loss of long-range input does not correlate with cell loss in distant brain regions. Interneurons transplanted into the injury site receive orthotopic local and long-range input, suggesting the machinery for establishing distant connections remains intact even after a severe injury. Our results uncover a potential strategy to sustain and optimize inhibition after traumatic brain injury that involves spatial reorganization of the direct inputs to inhibitory neurons across the brain.

## Abstract (web summary)

Traumatic brain injury is often followed by changes in neural connectivity. This study describes how inputs to a population of inhibitory neurons change to favor local over long-range connectivity in a mouse model of traumatic brain injury.

## Introduction

Brain function relies on an extremely diverse group of inhibitory interneurons that control the input and output of local networks^1–3^. In the cerebral cortex, one of the largest populations of interneurons expresses the neuropeptide, somatostatin (SST)^4–6^. These cells inhibit dendrites and thereby regulate the integration of glutamatergic input to local principal neurons. This endows them with unique roles in shaping synaptic plasticity, learning, and memory^7–14^. However, SST interneurons are among the most vulnerable to cell death following a brain injury, and their loss has been well documented in experimental models of epilepsy, traumatic brain injury (TBI), and Alzheimer’s disease^15–19^, and in humans^20,21^. In the hippocampus, surviving SST interneurons receive more excitatory drive, form new inhibitory synapses onto glutamatergic neurons and even grow into territories they normally do not occupy^22–25^. This pattern of local circuit rewiring raises the question of whether brain damage reorganizes interneuron connectivity on a much larger scale.

To address this possibility in an unbiased manner, we took advantage of a retrograde monosynaptic rabies virus system and enhanced whole-brain tissue clearing techniques to create brain-wide maps of the direct input to SST interneurons in a mouse model of focal TBI. We found dramatic quantitative differences in both the local and long-range input to hippocampal SST interneurons at the injury site. However, there was no neuron loss within the distant input regions themselves, and the proportion of neuron subtypes targeting starter neurons was stable. To our surprise, we uncovered a similar pattern of circuit reorganization far away from the injury in the prefrontal cortex (PFC), which interacts with the hippocampus bidirectionally^26^ but was not directly damaged by the initial insult. Interneuron progenitors grafted into the lesioned hippocampus successfully established appropriate long-range connections; however, graft-derived interneurons retained the enhanced local input seen after TBI. Thus, our experiments provide new insights about large-scale circuit remodeling following brain injury and suggest that brain damage, even when focally restricted, has a far broader impact on neural circuit function across the entire brain than previously appreciated.

## Results

### Long-term loss of SST interneurons after TBI

**Fig. 1:**
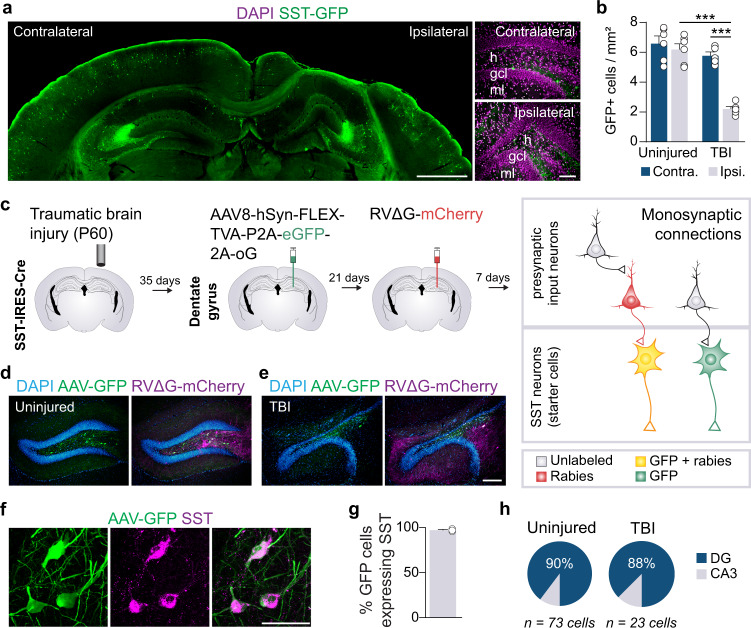
SST interneuron loss after focal TBI. **a** Coronal section 8 wks after TBI labeled for SST-GFP (green) and DAPI (magenta). h, hilus; gcl, granule cell layer; ml, molecular layer. Representative animal from *n* = 5 TBI mice **b** Quantification of SST-GFP interneurons in uninjured control and brain-injured animals. ***P = 1.62E-06, ipsilateral uninjured control versus ipsilateral TBI, ***P = 1.31E-05, contralateral TBI versus ipsilateral TBI; two-way ANOVA with Tukey’s post hoc test, *n* = 6 uninjured and 5 TBI mice. **c**. Schematic showing the two-virus experimental retrograde tracing strategy. **d, e** Dentate gyrus of an uninjured control (d) and CCI injured animal (**e**) labeled for DAPI (blue), AAV helper virus (green) and RVΔG-mCherry (magenta). Representative animals from *n* = 4 uninjured and 2 TBI mice. **f**. Coronal section of dentate gyrus labeled for AAV helper virus (green) and somatostatin (magenta). Representative animal from *n* = 3 uninjured mice. **g** Quantification of SST expression in neurons labeled with AAV helper virus; *n* = 3 mice. **h** Distribution of dual color-labeled starter cells in hippocampus; *n* = 73 cells from 4 uninjured controls, *n* = 23 cells from 2 animals with TBI. Error bars, s.e.m.; scale bars, 1 mm (**a**, left), 100 μm (**a**, right; **d** and **e**) and 50 μm (f). See also Supplementary Figs. 1, 2 and Supplementary Data 1. Source data are provided as a Source Data file.

Despite their important role in shaping local network activity and memory^11,14^, the precise brain-wide input to SST interneurons in the dentate gyrus has not been systematically defined. We first quantified SST+ neuron density during the chronic period after TBI using reporter mice that label nearly all SST interneurons with GFP^27^. A unilateral controlled cortical impact (CCI) injury was delivered to young-adult mice at P60 (1.0 mm impact depth, 3.5 m s^−1^ and 500 ms duration), and animals were processed for immunostaining eight weeks later. This period corresponds to a time when long-term neuropathology and behavioral phenotypes are well established. In all brain-injured mice, the lesion consisted of a cavity extending through the thickness of the neocortex and included substantial distortion and thinning of the principal cell layers in the hippocampus (Fig. 1a). We observed ~65% reduction in GFP+ cells in the hilus (Fig. 1b; Supplementary Data 1), consistent with short-term loss of SST interneurons reported in previous studies^17,18^.

### Visualization of input neurons to hippocampal SST+ interneurons

To label monosynaptic input to SST interneurons in the chronically injured brain, we used a genetically restricted two-virus approach to anatomically reveal their putative inputs (Fig. 1c-e). We first injected a Cre-dependent helper virus (AAV8-hSyn-FLEX-TVA-P2A-eGFP-2A-oG) into the dentate gyrus of adult SST-Cre mice 4 weeks after TBI. This helper virus provides the receptor that allows EnvA-coated rabies virus to enter only Cre-positive neurons and glycoprotein for rabies virus to transfer retrogradely to monosynaptic input neurons. Three weeks later, we injected a G-deleted and EnvA-pseudotyped rabies virus coding mCherry into the same location (RVΔG-mCherry). Because SST+ interneurons in the dentate gyrus are exclusively located in the hilus, we targeted this region for virus injection. After 7 days, we identified neurons that were positive for both fluorescent reporters at the injection site (starter cells) and neurons infected with the rabies virus were tagged with mCherry (pre-synaptic input neurons). Substantial numbers of neurons in different brain areas were labeled by rabies virus (Supplementary Fig. 1).

To confirm the specificity of virus labeling, we performed immunostaining against SST at the injection site. We found 97% of the GFP-labeled neurons were SST+ (152 of 156 cells, *n*  =  3 mice) (Fig. 1f, g). To evaluate the potential leakage expression of the virus, we performed a series of control experiments. To confirm the dependence of Cre recombination, we injected AAV8-hSyn-FLEX-TVA-P2A-eGFP-2A-oG helper and RVΔG-mCherry virus into Cre- littermates. No neurons were labeled anywhere in the brain (Supplementary Fig. 2a). In Cre+ animals, GFP+ neurons were only labeled at the injection site and no GFP+ neurons were found outside the injection site (Supplementary Fig. 2). In both control and brain-injured animals, starter cells were almost exclusively confined to the hilus (Fig. 1h, Supplementary Data 1). Only occasional starter cells were found in the adjacent CA3 region; no GFP+ cells were found in CA1, CA2, neocortex, or any other brain region.

### Whole-brain input to SST-positive neurons is reorganized by TBI

**Fig. 2:**
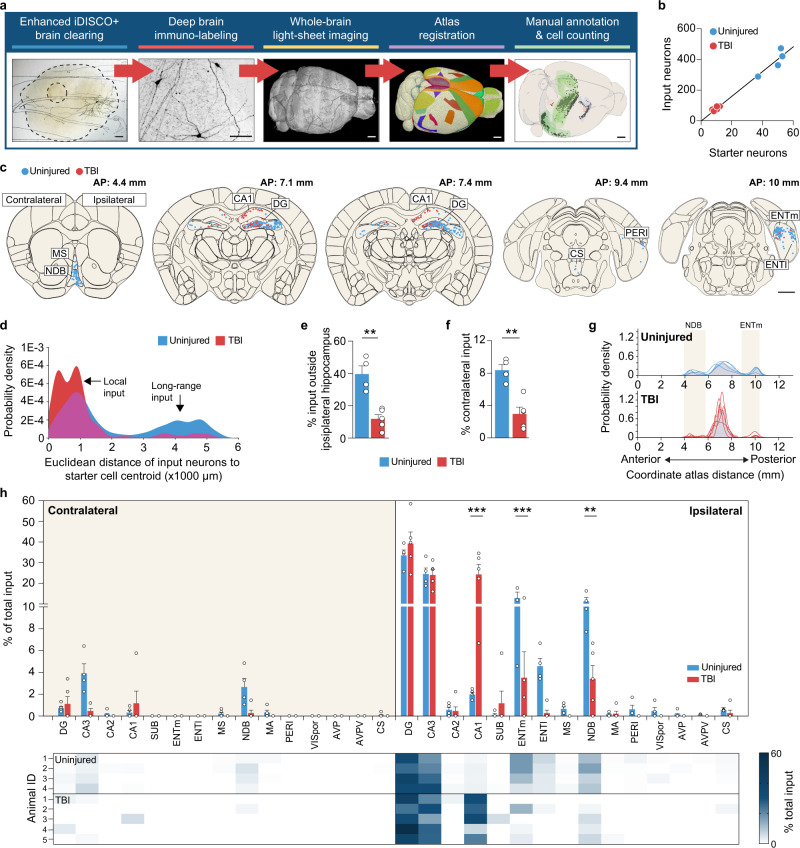
Reorganization of brain-wide input to hippocampal SST interneurons after TBI. **a** Experimental design for enhanced iDISCO+ brain clearing and whole-brain light-sheet imaging. **b** Linear regression analysis for number of starter cells and pre-synaptic input neurons in the whole brain (*n* = 4 uninjured controls, 5 TBI mice; *R*^*2*^ = 0.98). **c** Schematic coronal sections (250 μm) showing individual rabies-labeled cells registered in standardized atlas space for uninjured controls (blue) and brain-injured animals (red). One dot represents one neuron. *n* = 4 uninjured control and 5 TBI animals. A list of abbreviations is provided in Supplementary Data 2. **d** Gaussian kernel cell density plots showing pooled Euclidian distances of input neurons to nearest starter neuron centroid. **e**. Proportion of input neurons found outside ipsilateral hippocampus (DG, CA3, CA2, CA1). Uninjured: 39.6  ± 5.2%, *n* = 4 mice; TBI: 11.8 ± 2.7 %, *n* = 5 mice; **P = 1.5E-3; two-tailed *t*-test. **f** Proportion of input neurons found in the contralateral hemisphere. Uninjured: 8.4 ± 0.7%, *n* = 4 mice; TBI: 3.0 ± 0.8 %, *n* = 5 mice; **P = 2.0E-3; two-tailed *t*-test. **g** Gaussian kernel cell density plot of the whole-brain distribution of input neurons along the anterior-posterior axis. Grey shading represents the pooled population with individual lines representing each animal. Quantification is provided in Supplementary Data 3. **h** Proportion of input neurons found in each discrete brain area. ***P < 1.0E-15, uninjured versus TBI (CA1), ***P = 2.25E-04, uninjured versus TBI (ENTm), **P = 4.69E-03, uninjured versus TBI (NDB); two-way repeated-measures ANOVA with Bonferroni’s post-hoc test; *n* = 4 uninjured and 5 TBI mice. Quantification is provided in Supplementary Data 4. Error bars, s.e.m.; scale bars, 1 mm (except deep immunolabeling in a, 100 μm). See also Supplementary Figs. 3 to 7, Supplementary Data 5, and Supplementary Movies 1 and 2. Source data are provided as a Source Data file.

Next, we generated whole-brain maps of neurons sending monosynaptic input to SST interneurons using iDISCO+ brain clearing and whole-brain light-sheet imaging (Fig. 2a). One of the major challenges to these techniques has been accessing input neurons deep within the tissue^28^. Building on existing iDISCO+ protocols and recent advances in permeabilization chemistry^29,30^, we therefore modified the clearing conditions to achieve deep tissue immunolabeling in a traumatically injured brain. We made three key improvements in iDISCO+ sample preparation and imaging procedures (Supplementary Fig. 3). First, we incorporated an initial wash using N-methyl diethanolamine (MDEA) to decolorize residual unperfused blood, which absorbs light^31^. Second, we denatured the extracellular matrix using a concentrated guanidine hydrochloride solution prior to immunolabeling. Third, we incorporated an additional detergent (3-[(3-cholamidopropyl)dimethylammonio]-1-propanesulfonate, CHAPS) to the antibody diluent to enhance the penetration of antibodies deeper into the tissue. These optimizations enabled whole-brain immunolabeling without need for separating the brains into two separate hemispheres, as is commonly done. The raw data acquired from imaging were registered to the Allen Common Coordinate Framework (CCF) and cell positions were annotated and analyzed using tools from the BrainGlobe suite^32,33^ (Supplementary Fig. 4). As expected, the number of starter neurons was reduced in brain-injured animals (control: 48.0 ± 3.7 cells, TBI: 9.4 ± 0.9 cells, *P* = 9.45E-06, two-tailed *t*-test), consistent with the loss of SST interneurons. Starter cells were almost exclusively confined to the hilus (control: 92.8 ± 0.96% starter cells in dentate gyrus, TBI: 89.8 ± 3.2% starter cells in dentate gyrus; *P* = 0.3, Fisher exact test). Although the number of labeled neurons varied from animal to animal, there was a correlation between the number of input neurons and starter cells (Fig. 2b).

Whole-brain mapping of rabies-labeled neurons revealed input from 15 distinct brain regions (Fig. 2c, Supplementary Figs. 5, 6). The majority of input came from the dentate gyrus and CA3, as expected^24,34,35^ followed by the medial entorhinal cortex (ENTm), diagonal band nucleus (NDB) and lateral entorhinal cortex (ENTl). Of note, this pattern of input is different from previous retrograde tracing results in dentate granule cells, which receive considerably weaker projections from ENTm than ENTl and prominent input from mammillary nuclei^36,37^, as well as SST interneurons in CA1, which receive very little input from entorhinal cortex^38^. After TBI, there was a shift in the Euclidian distance of rabies-labeled neurons to the starter cell centroid (Fig. 2d; Supplementary Fig. 5), suggesting input neurons were substantially closer to starter cells after brain injury.

A higher proportion of local connections was confirmed when we analyzed the input to SST interneurons in greater detail. In control animals, approximately 40% of the input neurons were detected outside the hippocampus, and 8% of input neurons were located in the contralateral hemisphere. The proportions of these long-range inputs were significantly reduced in animals with TBI (Fig. 2e, f). Furthermore, we analyzed the probability of input neurons in 200 μm bins along the entire anterior-posterior (AP) axis, from 0 mm (olfactory bulb) to 13 mm (cerebellum) (Fig. 2g). This revealed significant increases in the percentage of input neurons between 6.8 mm to 7.4 mm at the level of hippocampus (6.8–7 mm, uninjured: 6.79 ± 0.79%, TBI: 18.12 ± 5.03%, *P* = 1.42E-11; 7–7.2 mm, uninjured: 7.15 ± 0.83%, TBI: 18.31 ± 4.01%, *P* = 3.02E-11; 7.2–7.4 mm, uninjured: 8.67 ± 1.70%, TBI: 16.72 ± 4.55%, *P* = 7.64E-06; two-way repeated-measures ANOVA with Bonferroni’s post-hoc test; Supplementary Data 3) and decreases at 10 mm to 10.2 mm at the level of ENTm (control: 7.48 ± 2.04%, TBI: 1.90 ± 1.03%, *P* = 1.33E-02; two-way repeated-measures ANOVA with Bonferroni’s post-hoc test; Supplementary Data 3). After TBI, massive input labeling was detected within the ipsilateral hippocampus, with a significantly higher proportion of input from area CA1 (Fig. 2h; Supplementary Data 4). However, distant brain areas providing the greatest proportion of input to SST interneurons in controls, such as ENTm and NDB, were found to have a significant reduction in input after TBI (Fig. 2h; Supplementary Data 4). To directly compare changes in the number of input neurons rather than the proportion of input, we calculated the convergence index for each animal, defined as the number of mCherry-labeled input neurons divided by the number of GFP- and mCherry-labeled starter cells. This analysis also revealed a significant increase in CA1 input, and decreases in long-range input from ENTm and NDB (Supplementary Fig. 7; Supplementary Data 5). Thus, there is a dramatic shift in both the number and relative proportion of local and long-distance input to hippocampal SST interneurons after TBI.

### Long-range input is proportionally stable, but local input is not

**Fig. 3:**
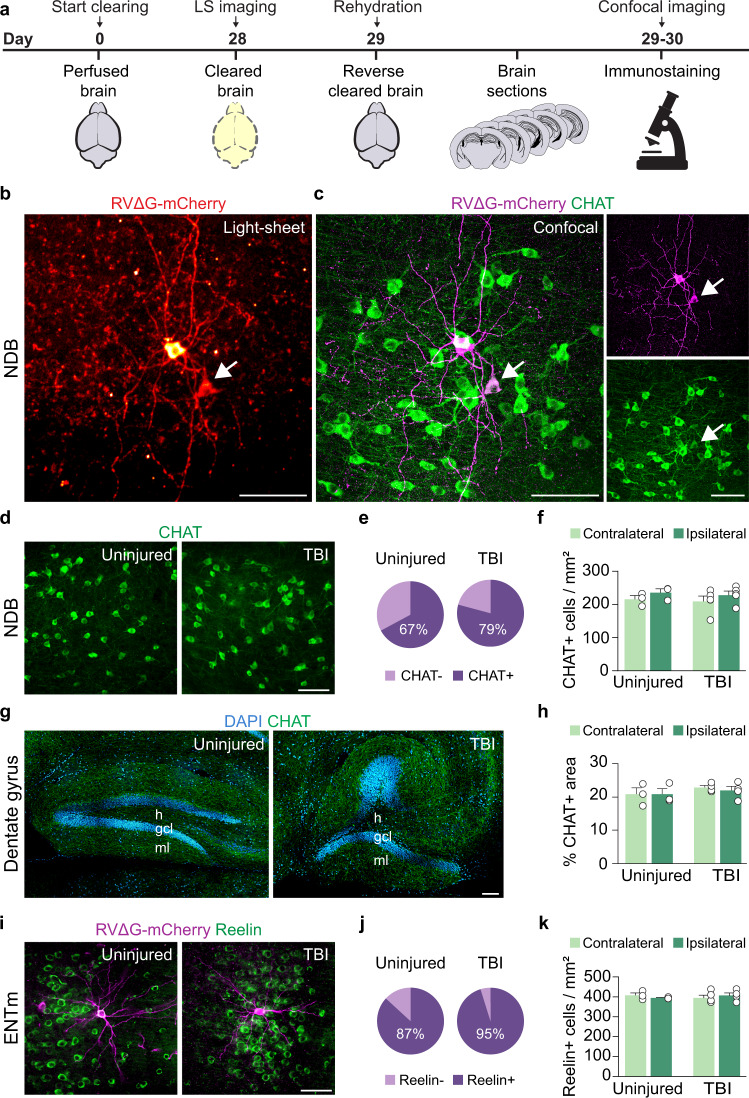
Distant brain regions remain structurally intact. **a** Schematic showing the experimental protocol for reverse brain clearing and immunolabeling. **b** Two rabies-labeled input neurons (red) identified in a sagittal optical section of NDB in an intact control brain. Representative animal from *n* = 3 uninjured controls. 50 μm maximum intensity projection obtained by whole-brain light-sheet imaging. **c** Sagittal section containing the same input neurons (magenta) labeled for CHAT (green) after processing for traditional immunostaining and confocal imaging. Representative animal from *n* = 3 uninjured controls. Arrow indicates co-labeled cell. **d** NDB of uninjured control (left) and brain-injured animal (right) labeled for CHAT (green). Representative animals from *n* = 3 uninjured and 4 TBI mice. **e** Proportion of mCherry+ rabies-labeled input neurons that expressed CHAT. **f** Quantification of CHAT+ neuron density in ipsilateral and contralateral NDB in uninjured controls and brain-injured animals. *n* = 3 uninjured and 5 TBI animals. **g** Sagittal section of dentate gyrus in uninjured control (left) and brain injured animal (right) labeled for CHAT (green) and DAPI (blue). Representative animals from *n* = 3 uninjured and 4 TBI mice. h, hilus; gcl, granule cell layer; ml, molecular layer. **h**. Quantification of CHAT+ axon density in ipsilateral and contralateral dentate gyrus in uninjured controls and brain-injured animals. *n* = 3 uninjured and 4 TBI animals. **i** ENTm of uninjured control (left) and brain-injured animal (right) labeled for reelin (green) and mCherry (magenta). Representative animals from *n* = 3 uninjured and 5 TBI animals. **j** Proportion of mCherry+ rabies-labeled input neurons that expressed reelin. **k** Quantification of reelin+ cell density in ipsilateral and contralateral ENTm. *n* = 3 uninjured and 5 TBI animals. Error bars, s.e.m.; scale bars, 100 μm. See also Supplementary Fig. 8 and Supplementary Data 1. Source data are provided as a Source Data file.

The loss of long-range input after TBI could result from a loss of neurons at distant sites or a loss of anatomical connections. To test this, and to characterize the neurochemical identities of the input to hilar SST interneurons, we developed a method for rehydrating the same brains used for light-sheet imaging and processing them for traditional double-immunofluorescence immunostaining (Fig. 3a-c). Because long-range input to hilar SST interneurons arrives primarily from basal forebrain and ENTm, we quantified neuron populations in these areas.

The basal forebrain contains multiple cell types that project long distances via the fimbria/fornix pathway to the hippocampus, including cholinergic, glutamatergic and GABAergic neurons^39^. In both control and injured animals, the absolute majority of NDB input neurons to hilar SST interneurons expressed choline acetyltransferase (CHAT) (control: 67.1 ± 6.8%, *n* = 3 mice; TBI: 79.2 ± 12.5%, *n* = 4 mice; *P* = 0.99, Fisher’s exact test; Fig. 3d, e). This is different from inhibitory interneurons in CA1, which primarily receive GABAergic input from the basal forebrain^38^. We did not find a difference in the density of CHAT+ neurons in NDB between control and brain injured animals (Fig. 3f), suggesting CHAT+ neurons were not reduced in the basal forebrain after focal TBI.

Because neurons in NDB and ENTm project directly into the hippocampus, their axons are almost surely damaged by TBI. Therefore, we next examined whether the density of cholinergic projections in the hippocampus were affected by TBI. In both control and injured animals, there was a dense plexus of CHAT+ axonal processes innervating every layer of hippocampus, even at the injury epicenter (Fig. 3g, h). We did not detect a difference in CHAT expression between groups (control, ipsilateral: 20.8 ± 1.9%, control, contralateral: 20.8 ± 1.7%, *n* = 3 mice; TBI, ipsilateral: 22.9 ± 0.6%, TBI, contralateral: 22.0 ± 1.2%, *n* = 4 mice; *P* = 0.96, two-way ANOVA). These results demonstrate the reduction in CHAT+ input neurons was not accompanied by a general loss of CHAT+ afferents in the hippocampus after TBI.

This result was unexpected, because a loss of CHAT immunoreactive neurons has been reported in basal forebrain of diffuse injury models^40,41^. To rule out the possibility that cell quantifications were influenced by rabies circuit mapping or brain clearing procedures, we examined a second, independent cohort of control and brain injured animals that did not undergo these procedures (*n* = 6 controls, *n* = 4 TBI animals). In this replication experiment, we also found similar numbers of CHAT+ neurons in NDB of age-matched control and TBI mice (Supplementary Fig. 8).

In both control and brain injured animals, input neurons in ENTm were found almost exclusively in layer II (Fig. 3i). Approximately 90% of these cells co-expressed reelin and had large multipolar stellate cell morphologies (control: 86.8 ± 0.34%, *n* = 3 mice; TBI: 95.2 ± 4.8%, *n* = 3 mice; *P* = 0.99, Fisher’s exact test; Fig. 3j). These results are consistent with prior studies showing that reelin-expressing stellate cells in ENTm give rise to the main associational glutamatergic pathway known as the perforant path that projects to the dentate gyrus, CA3 and CA2 regions of hippocampus^42–44^. We did not find a difference in the density of reelin+ neurons in ENTm between control and brain injured animals (Fig. 3k), similar to our results in basal forebrain. Together, our results demonstrate there is a reduction in the amount of input to hilar SST interneurons from both of the major distant brain regions innervating dentate gyrus after focal TBI, but these distant areas remain structurally intact.

**Fig. 4:**
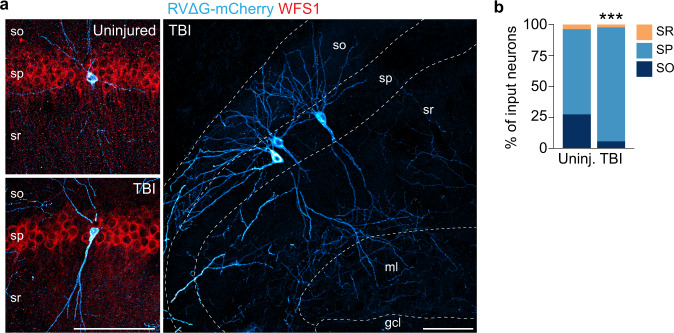
Identification of CA1 input neurons. **a** CA1 in uninjured control (top) and brain injured animal (bottom) labeled for rabies (blue) and WFS1 (red). Representative animals from *n* = 3 uninjured and 4 TBI mice. so, stratum oriens; sp, stratum pyramidale; sr, stratum radiatum; ml, molecular layer; gcl, granule cell layer. **b** Proportion of input neurons found in each layer of CA1. ***P = 1.29E-07, uninjured versus TBI, two-sided Chi-squared test. *n* = 45 cells from 3 uninjured controls, 183 cells from 4 TBI mice. Scale bars, 100 μm. See also Supplementary Data 1. Source data are provided as a Source Data file.

Input from CA1 increased more than 10-fold after TBI. To identify which CA1 neuron types provide input to hilar SST interneurons, we assessed the laminar position of CA1 input neurons in the same reverse-cleared tissue (Fig. 4). In controls, ~65% of CA1 input neurons were positioned in the pyramidal cell layer. These neurons had morphological features of pyramidal neurons and expressed WFS1, a marker of CA1 pyramidal neurons (Fig. 4a). A smaller portion of CA1 input neurons were found outside the pyramidal layer and did not express WFS1; these are putative interneurons. In brain injured animals, there was a significant increase in the proportion of input neurons positioned in the pyramidal cell layer (control: 64.5 ± 14.0%, *n* = 3 mice; TBI: 92.1 ± 5.1%, *n* = 4 mice; *P* = 1.29E-07, Chi-square test; Fig. 4b). Robust projections from these neurons could be clearly seen entering dentate gyrus (Fig. 4a). Therefore, there is an increase in local CA1 input to hilar SST interneurons after TBI, and CA1 pyramidal neurons are the primary source of this new input.

### Whole-brain connectivity is reorganized in PFC

Having observed dramatic brain-wide reorganization of hippocampal SST interneuron circuitry, we next asked whether the input to SST neurons was also rewired far away from the injury. The PFC is a critical higher-order limbic site that is central to memory retrieval and decision making and receives a highly diverse pattern of inputs spanning the entire brain, including direct input from hippocampus^45^. At 24 hrs after injury, fluoro-jade C staining revealed degenerating neurons in the hippocampus and neocortex at the injury epicenter, but no labeled cells were detected at distant sites, such as PFC, entorhinal cortex, basal forebrain or thalamus (Supplementary Fig. 9). This result is nearly identical to previous reports in this model^46^. We also did not find a difference in SST+ neuron density in PFC eight weeks after TBI (Supplementary Fig. 10; Supplementary Data 1). These results are consistent with producing a highly focal contusive brain injury.

**Fig. 5:**
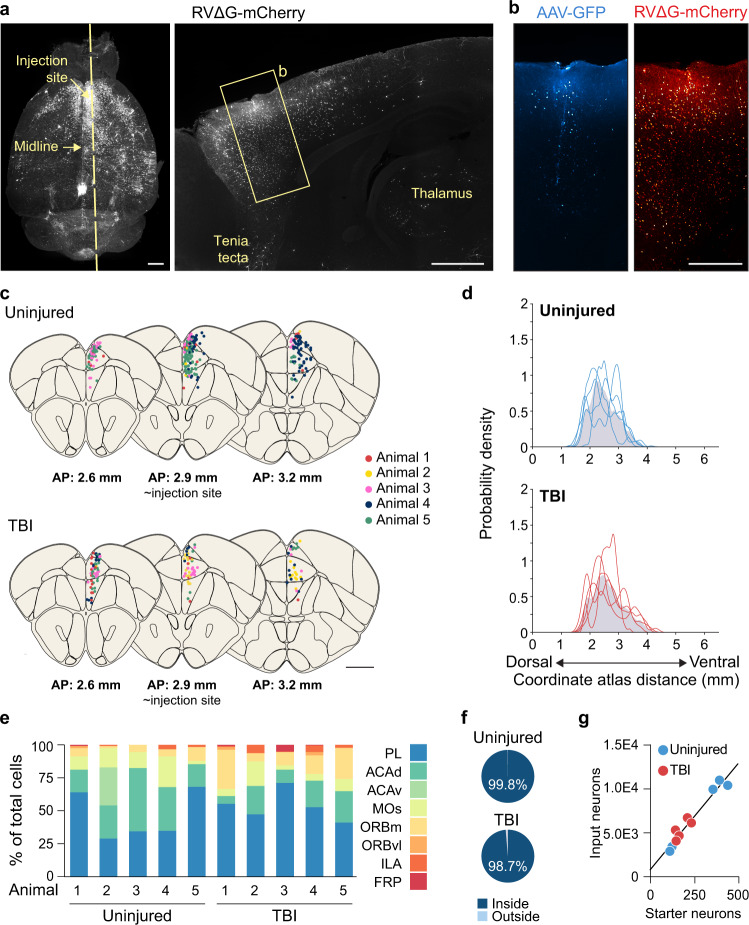
Distribution of starter neurons in PFC. **a** Left: Whole brain cleared using iDISCO+ and labeled for neurons providing input to PFC SST interneurons (white). Right: 100 μm sagittal optical section of the same brain showing rabies-labeled input neurons at the injection site. **b** Boxed region shown in (a) labeled for starter cells (blue) and input neurons (red). **c**. Schematic coronal sections (100 μm) showing individual starter cells registered in standardized atlas space for uninjured controls and brain injured animals. Each color corresponds to a different animal. One dot represents one neuron. *n* = 5 animals per group. **d** Gaussian kernel cell density plots of the whole-brain distribution of starter neurons along the dorsal-ventral axis. Grey shading represents the pooled population with individual lines representing each animal. **e** Regional distribution of starter cells in uninjured controls and brain injured animals. **f** Proportion of starter cells identified within PFC (PL, ACAd, ACAv, MOs, ORBm, ORBvl, ILA). **g** Linear regression analysis for number of starter cells and pre-synaptic input neurons (*n* = 5 mice per group; *R*^*2*^ = 0.95). Scale bars, 1 mm (a and c), 500 μm (b). A list of abbreviations is provided in Supplementary Data 2. See also Supplementary Fig. 9 to 12. Source data are provided as a Source Data file.

To generate whole-brain maps of the input to SST interneurons in PFC, we used the same two-virus rabies-based approach to label dual-color starter cells and mCherry-labeled input neurons in SST-Cre mice (Fig. 5a, b). For these studies, PFC was defined as including the following subregions based on a consensus drawn from the literature:^45^ secondary motor area (MOs), anterior cingulate areas dorsal and ventral (ACAd and ACAv), prelimbic area (PL), infralimbic area (ILA), orbital cortex medial, lateral and ventrolateral (ORBm, ORBl, and ORBvl). Injections were made into the ipsilateral hemisphere (that is, the injured side of the brain). We found 96.5% of the GFP-labeled neurons were SST+, and no neurons were labeled anywhere in the brain after injecting AAV8-hSyn-FLEX-TVA-P2A-eGFP-2A-oG helper and RVΔG-mCherry virus into Cre- animals (Supplementary Fig. 11). In both control and brain-injured animals, starter cells were almost exclusively located inside the PFC (Fig. 5c-f). Regional distributions of the starter cells were similar to what has previously been published for PFC^47^, and no differences were detected between groups in the dorsal-ventral location of the starter neurons (Fig. 5d; Supplementary Data 6) or layer distribution (Fig. 5e), indicating there was no major bias in the location of the starter cells. As expected, there was a correlation between the number of input neurons and starter neurons, but unlike hippocampus, the number of starter neurons were not reduced in brain-injured animals (control: *n* = 283.6 ± 69.5 cells; TBI: *n* = 178.8 ± 18.2 cells, *P* = 0.2, two-tailed *t*-test, *n* = 5 animals per group; Fig. 5g).

**Fig. 6:**
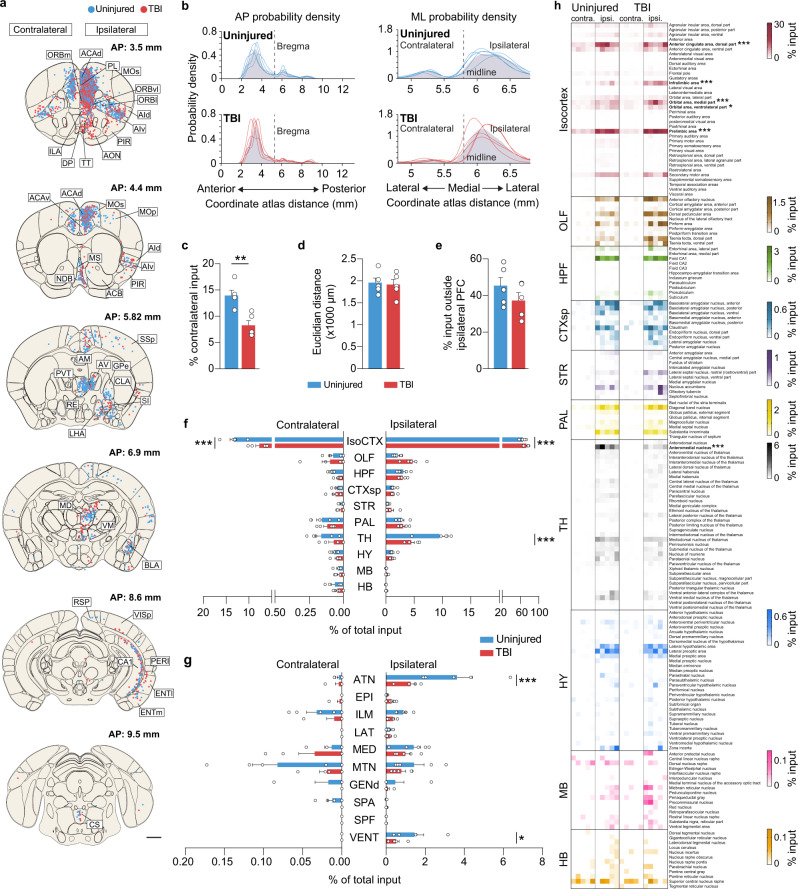
Input to SST interneurons in PFC is reorganized after TBI. **a** Schematic coronal sections (100 μm) showing individual input neurons registered in standardized atlas space for uninjured controls (blue) and brain injured animals (red). One dot represents one neuron. *n* = 5 animals per group. **b** Gaussian kernel cell density plots of the whole-brain distribution of input neurons along the anterior-posterior (AP) and medial-lateral (ML) axis. Grey shading represents the pooled population with individual lines representing each animal. Bregma, 5.3 mm; midline, 5.7 mm. **c** Proportion of input neurons found in contralateral hemisphere. Uninjured: 14.00  ± 1.02%; TBI: 8.22 ± 0.98 %; *n* = 5 mice per group; **P = 3.41E-03; two-tailed *t*-test. **d** Quantification of average Euclidian distance between starter cell centroid and input neuron positions. *n* = 5 animals per group. **e** Proportion of input neurons found outside ipsilateral PFC. *n* = 5 animals per group. **f** Proportion of total input arising from high-level brain regions. ***P = 2.84E-06, uninjured versus TBI (IsoCTX, contralateral), ***P = 4.46E-10, uninjured versus TBI (IsoCTX, ipsilateral), ***P = 4.12E-05, uninjured versus TBI (TH, ipsilateral); *n* = 5 mice per group; two-way repeated measures ANOVA with Bonferroni’s post hoc test. **g** Proportion of total presynaptic input arising from thalamic areas. ***P = 1.16E-11, uninjured versus TBI (ATN, ipsilateral), *P = 4.46E-02, uninjured versus TBI (VENT, ipsilateral); *n* = 5 mice per group; two-way repeated measures ANOVA with Bonferroni’s post hoc test. **h** Heatmap showing the proportion of input neurons identified in all discrete brain regions innervating PFC. ***P = 1.00E-15, uninjured versus TBI (ACAd, ipsilateral), ***P = 1.00E-15, uninjured versus TBI (ILA, ipsilateral), ***P = 1.00E-15, uninjured versus TBI (ORBm, ipsilateral), *P = 1.25E-02, uninjured versus TBI (ORBvl, ipsilateral), ***P = 1.00E-15, uninjured versus TBI (PL, ipsilateral), ***P = 1.18E-04, uninjured versus TBI (PL, contralateral), ***P = 1.07E-05, uninjured versus TBI (AM, ipsilateral); *n* = 5 mice per group; two-way repeated measures ANOVA with Bonferroni’s post-hoc test. Error bars, s.e.m.; scale bar, 1 mm. A list of abbreviations is provided in Supplementary Data 2. See also Supplementary Figs. 12 to 14, Supplementary Data 7 to 9, and Supplementary Movies 3 and 4. Source data are provided as a Source Data file.

Next, we quantified the input to SST interneurons in PFC. Whole-brain retrograde tracing revealed input from 178 distinct brain regions (Fig. 6a, Supplementary Figs. 12, 13). The majority of input was detected within the isocortex, especially in the ipsilateral hemisphere, followed by thalamus, hippocampus, and pallidum. These results are comparable to previous retrograde tracing results in control animals^47,48^. Within isocortex, PFC subregions provided the most prominent input; the agranular insular area and hippocampus area CA1 also provided prominent input. Analysis of the probability of input along the AP axis showed significant increases in the percentage of input neurons between 3.2 and 3.4 mm at the level of PFC in brain-injured animals (uninjured: 9.14 ± 1.14%, TBI: 12.06 ± 1.41%, *P* = 4.82E-03; two-way repeated measures ANOVA with Bonferroni’s post hoc test; Supplementary Data 7). Along the medial-lateral (ML) axis, we detected significantly higher proportion of input neurons between 0.2 and 0.5 mm lateral to midline in the ipsilateral hemisphere (5.9–6.0 mm, uninjured: 8.37 ± 1.38%, TBI: 11.38 ± 1.69%, *P* = 9.09E-07; 6.0–6.1 mm, uninjured: 10.20 ± 1.12%, TBI: 13.35 ± 0.81%, *P* = 2.08E-07; 6.1–6.2 mm, uninjured: 8.94 ± 0.61%, TBI: 11.90 ± 1.24%, *P* = 1.68E-06; two-way repeated-measures ANOVA with Bonferroni’s post-hoc test; Supplementary Data 7), and a significantly lower proportion of input neurons were found between 0.4 and 0.5 mm lateral to midline in the contralateral hemisphere (5.2 to 5.3 mm, uninjured: 3.22 ± 0.34%, TBI: 1.35 ± 0.32%, *P* = 3.51E-02; two-way repeated-measures ANOVA with Bonferroni’s post-hoc test; Fig. 6b, Supplementary Data 7). There was a significant decrease in the overall percentage of contralateral input to SST interneurons after TBI (Fig. 6c), similar to what we observed in the hippocampus. However, no differences were detected in the mean distance of input neurons to the starter cell centroid (Fig. 6d) or the overall percentage of input neurons located outside ipsilateral PFC (Fig. 6e).

Input neurons were initially grouped according to large functional divisions, i.e. isocortex, olfactory areas, hippocampal formation, cortical subplate, striatum, pallidum, thalamus, hypothalamus, midbrain and hindbrain. In brain injured animals, we found that SST interneurons in PFC received significantly greater percentage of total input from the ipsilateral isocortex as compared to controls, but the percentage of input neurons in the contralateral isocortex and ipsilateral thalamus were both reduced (Fig. 6f; Supplementary Data 8). In thalamus, anterior thalamic nuclei (ATN) and the ventral group of dorsal thalamus (VENT) showed a significantly lower proportion of input neurons after TBI (Fig. 6g). Whole-brain analysis of the input neurons in all discrete brain areas revealed that six of the seven areas with altered input after TBI were in the isocortex (Fig. 6h). Notably, not all PFC regions in the ipsilateral hemisphere had a significantly higher proportion of input neurons; input from ACAd and ORBvl were both significantly reduced after TBI. The proportion of input from contralateral PL was also reduced after TBI. Similar results were obtained by analyzing the convergence index for each animal (Supplementary Fig. 14; Supplementary Data 9). This pattern of enhanced local connectivity and reduced long-range input is similar to what we observed in brain injured hippocampus, suggesting that even in brain regions very far away from the injury site, the topographic organization of inhibitory neurons is dramatically rewired after a focal brain injury.

### Transplanted SST interneurons establish orthotopic brain-wide connections

**Fig. 7:**
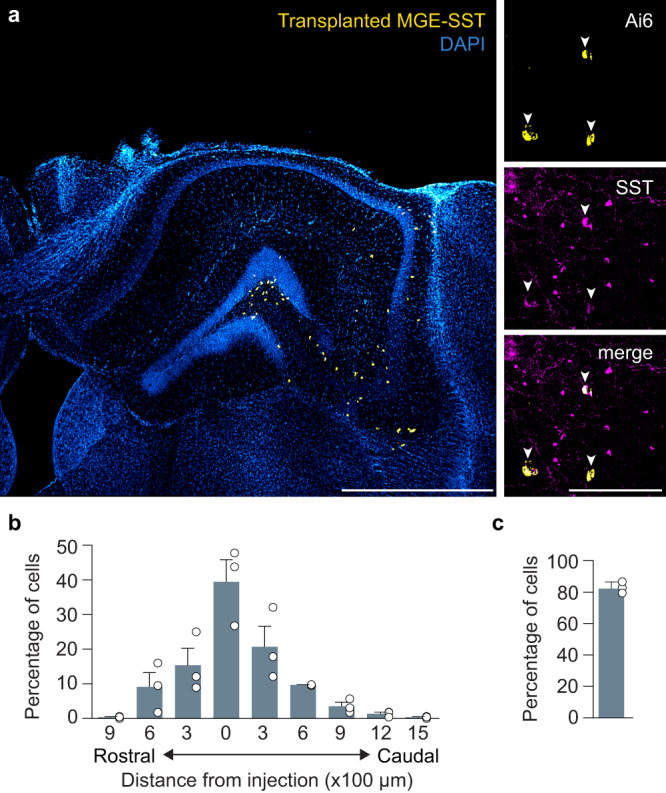
Transplanted SST interneurons integrate into brain-injured hippocampus. **a** Left: Coronal section of dorsal hippocampus five weeks after transplantation labeled for Ai6-expressing transplanted interneurons (yellow) and DAPI (blue). Representative animal from *n* = 3 mice. Scale bar, 1 mm. Right: Ai6-expressing neurons (yellow) co-labeled for somatostatin (magenta). Scale bar, 100 μm. **b** Distribution of transplanted SST interneurons 35 DAT, *n* = 3 mice. **c** Proportion of Ai6-expressing cells that expressed somatostatin, *n* = 3 mice. Error bars, s.e.m. See also Supplementary Fig. 15. Source data are provided as a Source Data file.

Grafts of embryonic-derived interneuron progenitors enable robust restoration of inhibition and are therapeutic in a wide range of acquired brain disorders, including epilepsy^49^, Alzheimer’s disease^50^ and TBI^51^. However, the circuit basis for this regeneration is unknown. Therefore, we tested whether interneuron progenitors are capable of establishing appropriate local and long-range connections in a damaged brain. For this purpose, we harvested GABA progenitors from the medial ganglionic eminence (MGE), the developmental origin of nearly all SST-expressing cortical interneurons^6^. Then, 7 days after TBI, we transplanted 3 × 10^4^ MGE cells into the ipsilateral hippocampus of C57BL/6 J mice at the injury epicenter. This corresponds to the period of maximal deafferentation after TBI^52^. We first examined grafts of SST interneurons harvested from E13.5 SST-Cre donor mice crossed with Ai6 reporter to allow for their visualization after transplantation. At 35 days after transplantation (DAT), transplanted interneurons were found throughout hippocampal subfields (*n*  =  3 animals) (Fig. 7a, b, Supplementary Fig. 15). The majority of Ai6-labeled cells expressed SST (83 ± 2.1%, Fig. 7c), confirming selective Cre expression in the SST population of transplanted MGE cells.

**Fig. 8:**
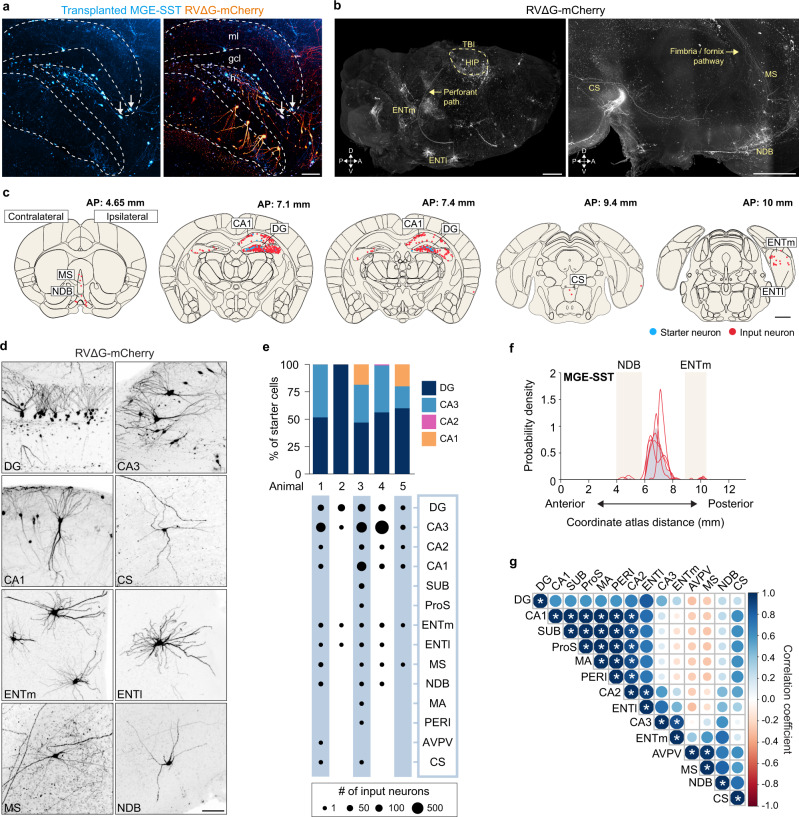
Transplanted SST interneurons receive local and long-range input. **a** A 100 μm sagittal optical section of dentate gyrus labeled for transplanted cells (blue) and input neurons (orange). White arrows, starter cells. h, hilus; gcl, granule cell layer; ml, molecular layer. **b** Left: whole-brain render of the entire ipsilateral hemisphere of the same animal shown in (a) labeled for input neurons (white). Dotted circle outlines the injury border overlying hippocampus (HIP). Right: Whole-brain render showing input neurons in superior central nucleus raphae (CS), medial septum (MS), and diagonal band nucleus (NDB). Representative animal from *n* = 5 mice. **c** Schematic coronal sections (250 μm) showing individual starter cells (red) and input neurons (blue) registered in standardized atlas space. One dot represents one neuron. *n* = 5 animals. **d** Maximum intensity projections (100 μm) of neurons providing input to transplanted SST interneurons in the intact injured brain. Representative animals from *n* = 5 mice. **e** Top: proportion of transplanted starter cells found in each layer of hippocampus. Bottom: bubble plot of brain areas containing rabies-labeled input neurons. **f** Gaussian kernel cell density plot of the whole-brain distribution of input neurons along the anterior-posterior axis. Grey shading represents the pooled population with individual lines representing each animal. **g** Correlation matrix of input to transplanted SST interneurons. Size and color, correlation coefficient. *P < 0.05, exact P-values are provided in Source Data file; two-sided Pearson’s correlation. Scale bars, 100 μm (**a, g**), 1 mm (**b, c**). A list of abbreviations is provided in Supplementary Data 2. See also Supplementary Fig. 16 and 17, Source Data and Supplementary Movie 5. Source data are provided as a Source Data file.

Next, we performed whole-brain mapping to identify local and long-range inputs to transplanted SST interneurons in the injured brain. For these experiments, donor cells were obtained from SST-Cre+ embryos that did not contain the Ai6 reporter. Transplants were performed 7 days after TBI, virus injections were made into the hippocampus at 8 weeks (AAV8-hSyn-FLEX-TVA-P2A-eGFP-2A-oG) and 11 weeks (RVΔG-mCherry) after injury, and animals were processed for brain clearing and analysis 7 days after the final virus injection. This period corresponds to a time when MGE transplantation shows robust therapeutic effects on memory and seizures in brain injured animals^51^. We found substantial numbers of input neurons across the brain labeled by rabies virus (Fig. 8a-c). Notably, input neurons were identified in 14 distinct brain areas, including all regions of the hippocampus, ENTm, ENTl, medial septum and NDB (Fig. 8c-f, Supplementary Fig. 16). The majority of input came from the hippocampus, including robust input from area CA3 and CA1. This pattern of local input was similar to what we observed in brain injured animals that did not receive transplants rather than controls (Supplementary Fig. 17). To determine whether regional inputs co-vary, we calculated Pearson’s correlation coefficients for each pair of input regions. This showed a significant positive correlation between several input regions including CA1, CA2, magnocellular nucleus (MA), prosubiculum (ProS), subiculum (SUB), and perirhinal cortex (PERI), indicating that when transplanted neurons receive input from one region, they generally received input from the other correlated regions (Fig. 8g). Thus, transplanted SST interneurons received orthotopic input patterns, but transplanted cells showed enhanced local input seen after TBI.

## Discussion

Using a mouse model of focal TBI, we report a systematic assessment of large-scale circuit reorganization in a damaged brain. We mapped the brain-wide input to a single cell type with high therapeutic relevance, SST interneurons, in uninjured controls, after brain injury and after transplantation into brain-injured animals. Our data demonstrate that surviving SST interneurons gain new local input after TBI, but they are largely disconnected from distant brain regions. This occurred at two spatially distinct but interacting brain areas: the injury site in the hippocampus and far away from the injury in PFC, which was not directly damaged by TBI. Our observation of circuit reorganization far away from the injury was unexpected, because PFC was not directly damaged by TBI and results from silver staining experiments suggest interhemisphere projections do not degenerate^46^. Thus, post-traumatic circuit rewiring is not restricted only to damaged areas, but occurs broadly throughout the brain in response to focal injury. We further show that the diminished long-range connections did not result from cell loss in distant brain areas or a general reduction in axon collaterals projecting into the damaged hippocampus. Transplanted SST interneurons integrated robustly into brain-injured hippocampus and received local and long-range host input that resembled the connections of native-born interneurons. This is consistent with previous ex vivo functional studies documenting robust excitatory drive onto transplanted interneurons from the host brain^49,51,52^, although the source of these inputs was previously unknown.

TBI produces major structural and functional alterations in neural circuitry, resulting from progressive brain damage and secondary neuroplasticity responses, which develop over time^53,54^. Until recently, our understanding of how TBI damages the brain’s wiring diagram has been limited to standard neuroanatomy and slice electrophysiology approaches. These studies show principal neurons undergo initial deafferentation after TBI, possibly due to loss of input neurons^53–56^. This is followed by a progressive increase in the number of synaptic contacts and the formation of new excitatory circuits within injured areas of the brain^54,57–62^. Excitatory drive onto hippocampal SST interneurons, which normally receive sparse input from local principal neurons, is also increased after brain injury^24^. However, slice recordings are limited to resolving functional connections of a few neurons within local circuits, because they are surgically isolated from long-range afferent input. By comprehensively mapping brain-wide inputs to SST interneurons at cellular resolution, we found a significant enhancement of local input neurons to hilar SST interneurons after TBI, in agreement with prior slice electrophysiology studies. We additionally identified previously unknown sources of input in controls, such as a back-projection pathway from CA1 to hilar SST interneurons which is enhanced after TBI. Notably, CA1 interneurons are also capable of projecting to dentate gyrus in temporal lobe epilepsy models^25^, but these cells primarily innervate granule cells.

Previous functional connectivity studies assume that TBI shifts the brain away from a small-world architecture, defined as densely connected local networks linked by sparse long-range input that connects discrete brain areas^63^. In line with this concept, interhemispheric resting-state fMRI connectivity is reduced after TBI^64^, possibly a result of diffuse axonal injury^63^, and there is a temporary increase in hyper-connectivity of local networks that generally weakens over time^65^. While these methods are useful for general hypotheses about brain connectivity, they are unable to identify a precise structural basis for circuit dysfunction in TBI and are likely disproportionately influenced by excitatory neurons. Our circuit mapping data are not entirely consistent with this idea at the cell type level. In both hippocampus and PFC, we found the most prominent sources of long-range input to SST interneurons, including input from the contralateral hemisphere, were diminished after focal TBI, but local connections are chronically increased. Thus, TBI may permanently enhance small worldness of inhibitory circuits by increasing local network connections and making long-range input more sparse. There is support for this idea from human functional connectivity studies^66^.

There is obvious axon damage after CCI^46^, and this likely affects long-range input to the hippocampus. We cannot exclude the possibility that some of the structural reorganization we observed after TBI is influenced by damage to white matter tracts. However, axon damage alone cannot fully explain the long-range circuit changes we observed in brain-injured animals. In hippocampus, we found ~4 fold reduction in long-range input to SST interneurons that was not accompanied by cell loss in distant brain regions or their projections in the hippocampus (e.g., CHAT+afferents). This suggests input regions are not disconnected from SST interneurons by a general loss of input neurons or axotomy. Rather, inputs may be added or removed in a cell-type-specific manner. It is possible that long-range afferents are selectively damaged by CCI but remain otherwise intact. For example, it is well known that TBI disrupts axonal transport mechanisms^67^, and this could disproportionately affect retrograde transport of rabies into long-range pre-synaptic input neurons. However, our finding that transplanted interneurons are capable of establishing long-distance connections at all suggests that the potential for re-growing these diminished inputs was retained in all brain-injured animals. We found similar increases in local input and decreases in long-range input to SST interneurons in PFC. Unlike hippocampus, these projections are not directly damaged and do not degenerate after CCI^46^. Of particular interest is the anteromedial thalamus, which was the only thalamic area with altered input neurons after TBI. Anterior thalamus (ATN, comprised of anteromedial, anteroventral and anterodorsal subdivisions) provides a subcortical circuit supporting memory and spatial navigation^68^, behaviors that are profoundly affected in rodent models of TBI. It is possible that excessive activity in damaged hippocampus could lead to downstream changes in PFC (e.g., via the hippocampus-ATN-PFC circuit). Alternatively, seizures are well documented in this injury model and could lead to circuit reorganization across the entire brain. Nevertheless, our results suggest that focal TBI leads to widespread remapping of inputs to SST interneurons across the brain regardless of whether there was direct injury or cell loss.

With far fewer inhibitory neurons in the damaged hippocampus, TBI puts extraordinary demands on surviving interneurons. There are three physiological features of SST interneurons that might explain why a loss of long-range input could reflect a compensatory response to damage. First, SST interneurons recieve strongly facilitiating excitatory synaptic input and have other membrane properties that allow these cells to be activated by a high-frequency burst from just one pre-synaptic neuron^69,70^. In hippocampus, gaining input from local principal neurons and losing ENTm input may help stabilize local network activity after TBI^71^. Second, muscarinic-mediated depolarization can produce prolonged spiking in SST interneurons^72^. Given the rich local innervations after TBI, the loss of CHAT input from basal forebrain could reflect a strategy to balance the excitatory drive to this important cell type in the injured brain. Third, even a single dendrite-targeting interneuron can control spike generation in cortical principal neurons^7^. Thus, a shift from feed-forward to feedback inhibition to dendrites – through the loss of long-range input and enhanced local back-projections – may reflect a potential strategy to gate input integration and maintain the sparse activation of dentate granule cells that enables memory and prevents seizures^73,74^. Alternatively, increased local input may synchronize SST interneurons, support patterns of pathological activity or impede the coordination between discrete brain areas. Further work combining in vivo neurophysiology with selective manipulation of hippocampal cell types will be required to clarify these possibilities.

The ability of transplanted SST interneurons to incorporate structurally into the damaged hippocampus was striking given the dramatic reorganization of inhibitory circuits across the injured brain. Although host-donor cell connectivity has been broadly documented^75,76^, the precise anatomical input to individual neuron types has not. Cell-type specificity is an important consideration, especially for understanding the circuit basis of disease. Despite massive reactive plasticity in the damaged brain, we found that transplanted interneurons receive highly orthotopic input that is cell-type-specific rather than region-specific. We propose that the beneficial effects of interneuron transplantation seen in various preclinical disease models are driven by the precise integration of interneuron precursors into host brain circuits. This view is supported by a large body of evidence reporting the general structure and function of transplanted interneurons closely resemble their native-born counterparts^49,51,52,77–79^ as well as recent DREADD-inactivation and VGAT loss-of-function studies demonstrating that therapeutic effects are linked to the electrophysiological integration of the transplanted interneurons^51,80^. An alternate view suggests interneuron precursors form only weak contacts with the host brain^81^ and work indirectly by releasing rejuvenation factors that modify host brain circuits^82^. Yet detailed electrophysiological studies consistently report strong synaptic connections^51,52,83,84^ and direct evidence for a circuit rejuvenator remains to be identified. In the current study, we were unable to directly test synaptic connections among transplanted interneurons, because it was not possible to distinguish starter cells from AAV-labeled SST neurons that received rabies via retrograde transport. However, the large majority of local input neurons were putative principal neurons, not inhibitory neurons (based on morphology and laminar location). This is consistent with a robust literature on MGE transplantation using EM ultrastructural analysis, neuroanatomy, and patch-clamp electrophysiology that demonstrates most synaptic connections are with principal cells of the host brain^51,52,77,81–85^.

The pattern of synaptic circuit rewiring after TBI is complex. Our results suggest that focal brain damage reorganizes inhibitory circuits on a global scale. We expect this experimental approach will serve as a useful framework for considering whole-brain analyses of network dysfunction in TBI and related brain disorders.

## Methods

### Animals

All animal procedures were performed under Institutional Animal Care and Use Committee (IACUC) approval by the University Laboratory Animal Resources at the University of California, Irvine and adhered to National Institutes of Health Guidelines for the Care and Use of Laboratory Animals. Experiments were performed on adult mice of both sexes maintained in standard housing conditions on a 12 h light/dark cycle with food and water provided *ad libitum*. For SST cell quantifications, we used GIN mice maintained on a FVB background (Jax Stock No: 003718). For rabies circuit tracing, we used Sst-IRES-Cre mice maintained on a C57BL/6 J background (Jax Stock No: 018973). We used C57BL/6 J mice (Jax Stock No: 000664) for fluoro-jade C experiments. For cell transplantations, embryonic donor tissue was produced by crossing Sst-IRES-Cre J mice with C57BL/6 J mice (Jax Stock No: 000664) or Ai6-ZsGreen reporter mice (Jax Stock No: 007906); host mice were C57BL/6 J mice (Jax Stock No: 000664).

### Experimental Design

Experiments were performed on male and female littermates between P55 and P139. Upon weaning, animals were coded and randomly assigned into uninjured (naïve control), TBI or MGE-injected treatment groups. Brain injured mice and age-matched controls were housed together (2–5 animals per cage) within a temperature- (21–22 °C), humidity- (40–51%), and light- (12 h light:dark cycle) controlled vivarium. The order of injury, virus injection and cell transplantation was also randomized. Blinding was not possible due to the presence of an injury in TBI treatment group. CHAT immunostaining experiments were replicated using a separate, independent cohort of control and brain-injured animals. No other replication studies were performed.

### Brain injury

CCI injury was performed on adult male and female mice at P55^18^. Briefly, mice were anesthetized by 2% isoflurane inhalation and placed in a custom stereotaxic frame. The skull was exposed by midline incision, and a 4–5 mm craniotomy was made ~1 mm lateral to the sagittal suture and centered between bregma and lambda. The skull cap was removed without damaging the underlying dura. The contusion device consisted of a computer-controlled, pneumatically driven impactor fitted with a beveled stainless-steel tip 3 mm in diameter (Precision Systems and Instrumentation; TBI-0310). Brain injury was delivered using this device to compress the cortex to a depth of 1.0 mm at a velocity of 3.5 m s^−1^ and 500 ms duration. The incision was sutured without replacing the skull cap, and the animal was allowed to recover on a heating pad. A qualitative postoperative health assessment was performed daily for 7 d after TBI and periodically thereafter. All animals that received surgery were treated with buprenorphine hydrochloride (Buprenex; 0.05 mg/kg, delivered i.p.) at the time of surgery and 24 h later. All brain-injured mice survived and remained otherwise healthy until the day of experimentation.

### Virus injections

AAV8-hSyn-FLEX-TVA-P2A-GFP-2A-oG with a titer of 1.2 × 10^13^ genome copies/mL was obtained from the GT3 Core Facility of the Salk Institute and diluted to a titer of 2.4 × 10^11^genome copies/mL in sterile 0.9% NaCl prior to use, to prevent impairments in tracing performance^86^. RVΔG-mCherry was produced as previously described^87^ with a titer of 5 × 10^9^ infectious units/mL. Virus was front loaded into beveled glass micropipettes (40 μm tip diameter, Wiretol 5 μl, Drummond Scientific) and injected into the brains of adult uninjured control and brain injured mice at a rate of 15 nL min^–1^ and the needle was left in place for 5 min before retraction. Target coordinates were first verified in a series of preliminary injection studies into control and brain injured mice to determine ideal target locations (e.g., hilus) and the titer and volume of virus that could be delivered into hilus or PFC without leakage into nearby regions. AAV injections (200 nL) were made into hilus of dentate gyrus at the following stereotaxic coordinates: anterior-posterior (AP) −2.0 mm, medial-lateral (ML) 1.30 mm, dorsal-ventral (DV) −1.9 mm. In separate cohort of animals, injections were made into prelimbic cortex: AP 1.8 mm, ML 0.35 mm and DV −1.4 mm. RVΔG-mCherry (100nL) was injected 3 weeks later at the same location.

### Tissue clearing and whole-brain immunostaining

Mice were transcardially perfused with 0.1 M PBS containing 1uL/mL of 10 mg/mL heparin sodium (Serva cat no. 24590.01) followed by 4% PFA in 0.1 M PBS. Samples were postfixed overnight in 4% PFA in 0.1 M PBS. Subsequent steps were performed in 5 mL centrifuge tubes (Eppendorf cat. No 0030119401) with 0.01% sodium azide added to each solution. First, samples were decolorized in 10% 3-[(3-Cholamidopropyl)dimethylammonio]-1-propanesulfonate (CHAPS, Anatrace cat. no. C316S) and 25% N-methyl diethanolamine (MDEA, Alfa-Aesar cat. no. L15712) in 0.1 M PBS for 48 h at 37 °C with nutation. Samples were then washed in 0.1 M PBS for 24 h, dehydrated in a methanol/water gradient (20%, 40%, 60%, 80%, 100%, 100%) for 1 h each and delipidated in 2:1 DCM:MeOH overnight. The next day, samples were washed twice in 100% MeOH for 4 hr to remove DCM:MeOH and bleached in 5% H_2_O_2_ in 80% MeOH overnight at 4 °C without shaking. Samples were then rehydrated in 60% MeOH, 40% MeOH, 20% MeOH, 0.1 M PBS and PTx.2 for 1 hr, incubated in 4 M guanidine hydrochloride (Alfa-Aesar cat. no. A13543-30) and 1% CHAPS in 0.1 M PBS for 24 h and washed overnight in 0.1 M PBS with three solution changes. Next, samples were permeabilized in 10% CHAPS/25% MDEA in 0.1 M PBS overnight at 37 °C with nutation, incubated in 3%NDS/10%DMSO in PTx.2 with nutation at 37 °C overnight. Primary antibody incubations were performed in heparinized 0.1 M PBS containing 0.2% Tween-20 (PTwH) containing 0.25% CHAPS, 3% NDS and rabbit anti-DsRed (1:1000) and chicken anti-GFP antibodies (1:1000) for 7 days at 37 °C with nutation. Samples were then washed in PTwH overnight on an orbital shaker (115 RPM) at room temperature with five solution changes. Secondary antibody diluent was prepared in PTwH containing 0.25% CHAPS, donkey anti-rabbit 546 (1:1000) and goat antichicken 647 (1:1000), syringe filtered at 0.2 μm and incubated for 7 days at 37 °C with nutation before washing in PTwH overnight with five solution changes and shaking at room temperature. The next day, samples were dehydrated in increasing methanol/water gradients (20%, 40%, 60%, 80%, 100% for 1 h each) and allowed to sit in 100% MeOH overnight at 4 °C. Samples were then washed in 2:1 DCM/MeOH for 3 hrs, followed by two 100% DCM washes for 15 min each and cleared in dibenzyl ether (DBE) overnight at 4 °C. DBE was changed four additional times before imaging for refractive index matching. A step-by-step protocol for whole-brain immunostaining can be found at: https://github.com/roberthuntlab/clearedbrainanalysis.

### Light-sheet imaging

Cleared samples were mounted in a 3D-printed sample holder in a custom-built imaging chamber with the same lot of DBE solution used for refractive index matching and imaged using a Zeiss Z1 light-sheet microscope with Zeiss Zen software. Samples were imaged in the sagittal orientation with single-sided illumination using a x5/0.1 illumination objective and a x5/0.16 detection objective at 0.91 μm/pixel resolution with 4.97 μm step size. mCherry-labeled input neurons were imaged using a 561 nm laser coupled to a 575–625 nm BP filter. GFP and auto-fluorescence channels were acquired simultaneously with 488 nm and 638 nm laser lines coupled to 505–545 nm BP and 660 nm LP filters. Laser power was set to 40% intensity for all laser lines with 200 ms exposure. Tile overlap was set to 8%.

### Whole-brain 3D image registration and annotation

Raw data (.czi) were converted into hierarchical format (.ims) using Imaris File Converter 9.1 (Bitplane). The 561 nm channel data was downsampled by a factor of two in each dimension and exported as numpy arrays (.npy) using custom Python scripts. Individual tiles were stitched non-rigidly using WobblyStitcher^88^. Stitched arrays were exported as.tif series with a background subtraction value determined for each animal. Individual cell positions were manually annotated using cellfinder^33^. Image stacks were downsampled to 10 μm resolution and registered to the Allen Reference Atlas^89^ using brainreg, a Python port of aMAP^90^. Atlas boundaries were upsampled to the original high-resolution image in Image J, and image planes containing cells were inspected for accuracy. To correct for whole-brain registration error, pairs of correspondence points were manually marked where atlas boundaries diverged from anatomical landmarks. Average vector length was calculated, and annotated cell positions were linearly transformed based on the correspondence point vector length. Schematics of the anatomical position of starter cells and input neurons were plotted using brainrender^32^. Coronal atlas plates were rendered at selected positions along the anterior/posterior axis and individual cells positions ± 125 μm (hippocampus tracing) or ± 50 μm (PFC tracing) to the selected atlas plate.

### Whole-brain quantification

Registered cell positions for each animal were summarized using cellfinder^33^. Cell counts were combined for laminated cortical strucutures. Ipsilateral and contralateral cell counts were analyzed as separate regions. In one uninjured control (hippocampus injection), a small number of rabies-labeled cells were found near the injection tract in overlying neocortex. These cells were excluded from analysis as they were labeled by the needle injection, not trans-synaptic spread of virus. Each individual brain area used for quantification and their abbreviation within the Allen CCF atlas are provided in Supplementary Data 2. For Euclidian distance calculation, the starter cell centrioid was calculated by averaging the atlas coordinates of each registered starter cell and rounding the result to the nearest integer. Three dimensional Euclidian distance was calculated between the starter cell centriod and each pre-synaptic input neuron position using the following formula:

1 $$d=\sqrt{({x}_{2}-{x}_{1})+({y}_{2}-{y}_{1})+({z}_{2}-{z}_{1})}$$

where (*x*_2_,*y*_2_,*z*_2_) represents AP, ML and DV of the starter cell centriod, (*x*_1_,*y*_1_,*z*_1_) represents AP, ML and DV of each registered pre-synaptic cell position and *d* represents the length of the calculated distance vector. For analysis of AP and DV distances, cell counts were binned at 200 μm intervals and normalized to the total number of cells for each animal. For analysis of ML distance, cell counts were binned at 100 μm to produce an even number of bins for each hemisphere. Gaussian kernel density estimates were computed using seaborn python script, and bandwidth was set at 0.5.

### Reverse clearing

Cleared samples were removed from DBE and washed in DCM twice for 15 min followed by overnight incubation in 2:1 DCM/MeOH. Samples were then rehydrated in 100%, 80%, 60%, 40%, 20% MeOH/water and 0.1 M PBS for 1 hr each on an orbital shaker (115 RPM) at room temperature. Free-floating vibratome sections (50 μm) were cut using a vibratome at room temperature in 0.1 M PBS containing 0.05% Triton-X. A step by step protocol for reverse clearing can be found at: https://github.com/roberthuntlab/clearedbrainanalysis.

### Immunostaining

Mice were transcardially perfused with 4% paraformaldehyde (v/v) and free-floating vibratome sections (50 μm) were processed using standard immunostaining procedures^51^. Primary antibodies were as follows: chicken anti-green fluorescent protein (GFP; 1:1000; Aves, cat no. GFP1020), chicken anti-mCherry (1:1000; Abcam, cat no. ab205402), goat anti-choline acetyltransferase (CHAT; 1:500; Millipore, cat no. AB144P), mouse anti-reelin (1:500; Millipore, cat no. MAB5364, clone G10), rabbit anti-DsRed (1:1000; Clontech, cat no. 632496), rabbit anti-somatostatin (SST; 1:200; Santa Cruz, cat no. SC-7819) and rabbit anti-WFS1 (1:1000; Protein Tech, cat no. 11558-1-AP). All antibodies have been previously used for immunostaining analysis in brain. For secondary antibodies (1:1000, Life Technologies), we used Alexa 488–conjugated goat antibody to chicken IgG (cat. no. A11039), goat antibody to mouse IgG (cat. no. A11029), donkey antibody to goat IgG (cat. no. A11055); Alexa 546-conjugated goat antibody to rabbit IgG (cat. no. A11035), donkey antibody to rabbit IgG (cat. no, A10040), Alexa 594–donkey antibody to goat IgG (cat. no. A11058) and Alexa 647–conjugated goat antibody to chicken IgG (cat. no. A32933). Sections were then mounted on charged slides (Superfrost plus, Fisher Scientific) with Fluoromount-G containing DAPI. Confocal images were obtained with an Olympus FV3000 laser scanning microscope. Epifluorescent images were obtained using a Leica DM6 microscope with Leica LAS X software. Brightness and contrast were adjusted manually using Image J, as needed.

### Cell quantification in brain sections

Fluorescently labeled sections (50 μm) were imaged using a Leica DM6 microscope with a ×10 or ×20 objective or Olympus FV3000 confocal microscope with a ×20 or ×40 objective and counted using FIJI (ImageJ)^51^. All cells that expressed a fluorescent marker were counted in every sixth section through the entire brain (that is, 300 μm apart). All sections containing labeled cells were analyzed per animal and the values averaged to obtain a mean cell density (cells/mm^2^).

### Fluoro-jade C staining

Mice were transcardially perfused with 4% paraformaldehyde (v/v) and free-floating vibratome sections (50 μm) were processed for fluoro-jade C staining according to manufacturer instructions^91^. Briefly, brain sections were dried on gelatin-coated slides for 30 min at 50 °C. Slides were immersed in 1% NaOH in 80% ethanol for 5 min, 70% ethanol for 2 min, dH_2_O for 2 min, 0.06% potassium permanganate for 10 min, dH_2_O for 2 min, 0.00015% fluoro-jade C (Histo-Chem Inc., cat no. 1FJC) and 0.0001% DAPI in 0.1% acetic acid for 10 min, followed by three washes in dH_2_O for 1 min each. Slides were then dried at 50 °C for 5 min and cleared in xylenes before coverslipping with Eukitt mounting medium (Sigma, cat. no 03989).

### Tissue dissection and transplantation

Ventricular and subventricular layers of the MGE were harvested from E13.5 embryos. The time point at which the sperm plug was detected was considered E0.5. Embryonic MGE explants were dissected in Leibovitz L-15 medium, mechanically dissociated by repeated pipetting in L-15 medium and concentrated by centrifugation (3 min at 600 × *g*). Concentrated cell suspensions were front loaded into beveled glass micropipettes (50 μm tip diameter, Wiretol 5 μl, Drummond Scientific) and injected (3 × 10^4^ cells per injection) into the hippocampus of adult brain-injured mice 7 d after CCI injury. Cell injections were made into stratum radiatum of the CA3 subfield at the following stereotaxic coordinates: AP −2.0 mm, ML 2.45 mm, DV −1.8 mm.

### Statistical analysis

All statistical tests were performed with Graphpad Prism 9, R and Microsoft Excel. Sample sizes were based on previous publications^38,47,92^. For whole-brain quantitative analyses, the number of input neurons in each distinct brain area was normalized to the total number of input neurons detected in the whole brain (% input) or to the total number of starter cells (convergence index). Data were compared by two-tailed student’s *t*-test, one-way ANOVA followed by a Tukey’s post hoc test for multiple comparisons, two-way ANOVA followed by a Tukey’s post hoc test for multiple comparisons, two-way repeated-measures ANOVA followed by a Bonferroni’s post hoc test, Chi-Square analysis or Fisher’s exact test. UMAP analysis and Pearson correlation was computed in R. Data are expressed as mean±SEM, *n* = animals unless otherwise specified and significance was set at P < 0.05. For a complete list of statistical tests and results, see Supplementary Data 1–9 and Source Data.

### Reporting summary

Further information on research design is available in the Nature Research Reporting Summary linked to this article.

## Supplementary information

Supplementary Information

Description to Additional Supplementary Information

Supplementary Data 1-9

Supplementary Movie 1

Supplementary Movie 2

Supplementary Movie 3

Supplementary Movie 4

Supplementary Movie 5

Reporting Summary

## Data Availability

All data generated in this study are provided in the Supplementary Information and Source Data file. Source data are provided with this paper.

## Source data

Source Data

## Code availability

Custom Python scripts are available at: https://github.com/roberthuntlab/clearedbrainanalysis.
