# Supplementary material for: Brain-wide reconstruction of inhibitory circuits after traumatic brain injury
Source: Nat Commun. 2022 Jun 14;13:3417. doi: 10.1038/s41467-022-31072-2 (PMC9197933; doi:10.1038/s41467-022-31072-2)
Supplement: Supplementary file 1 — Supplementary Information [file 41467_2022_31072_MOESM1_ESM.pdf]

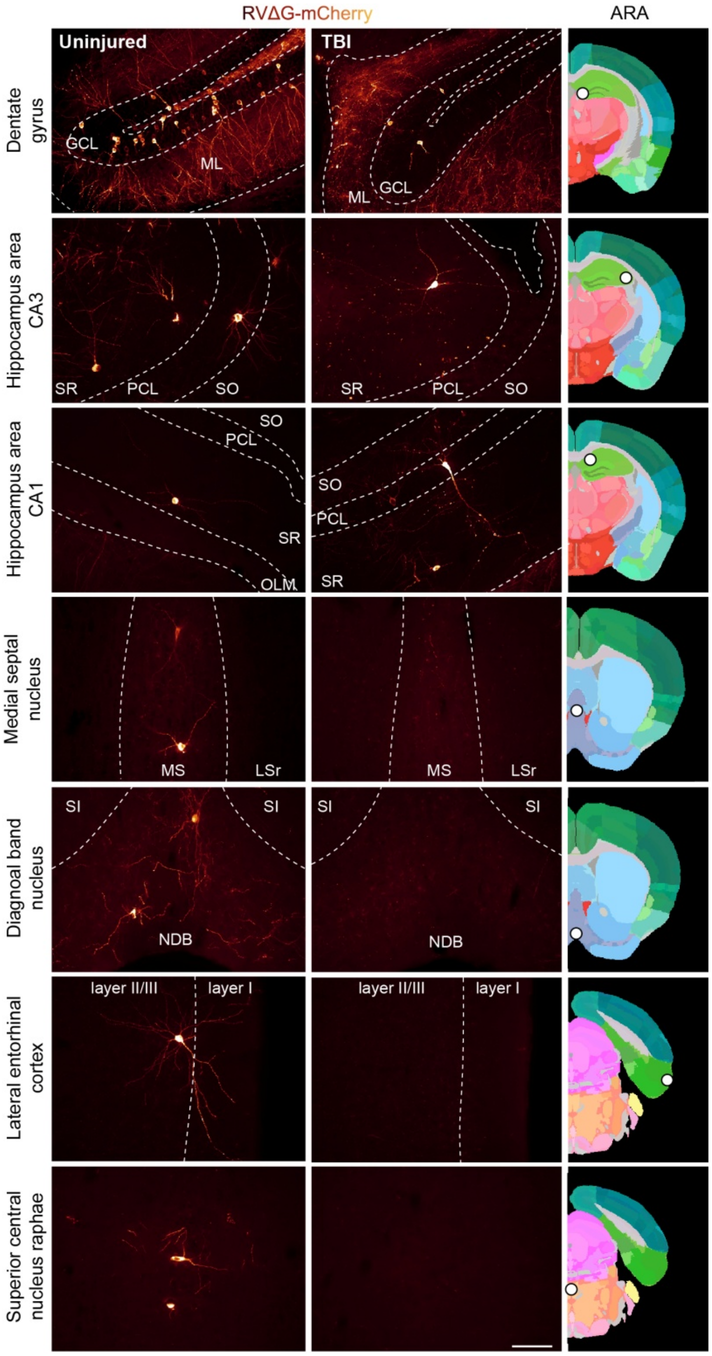

Supplementary Figure 1: Input neurons to hilar SST interneurons. Coronal sections labeled for input neurons (red) in an uninjured control (left) and 8 wks after TBI (right). Representative animals from *n* = 4 uninjured and 2 TBI mice. Image location is indicated by a white dot overlaid onto the corresponding atlas plate from the Allen Reference Atlas (ARA). GCL, granule cell layer; ML, molecular layer; SR, stratum radiatum; PCL, pyramidal cell layer; SO, stratum oriens; OLM, oriens lacunosum moleculare; MS, medial septal nucleus; LSr, lateral septal nucleus, rostroventral part; NDB, diagonal band nucleus; SI, substantia innominata. Scale bar, 100 µm.

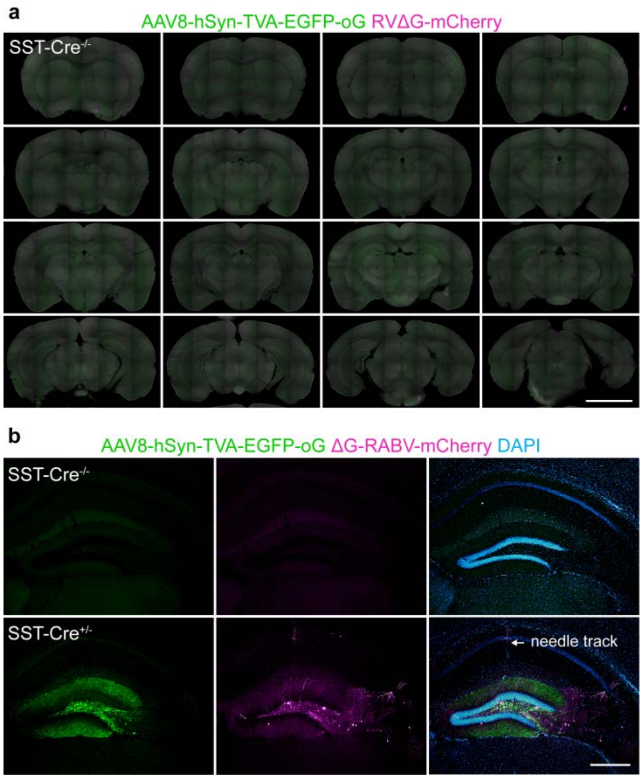

Supplementary Figure 2: Virus specificity in hippocampus. a. Serial coronal sections every 300 μm through the entire brain of an adult SST-Cre-negative mouse labeled for AAV helper virus (green) and RVΔG-mCherry (magenta). Representative animal from *n* = 3 mice. **b.** Coronal section of hippocampus labeled for helper virus (green) and rabies (magenta) in SST-Cre-negative mouse (top) and SST-Cre-positive mouse (bottom). Representative animal from *n* = 3 SST-Cre-negative and 4 SST-Cre-positive mice. Scale bar, 2 mm (a); 500 μm (b).

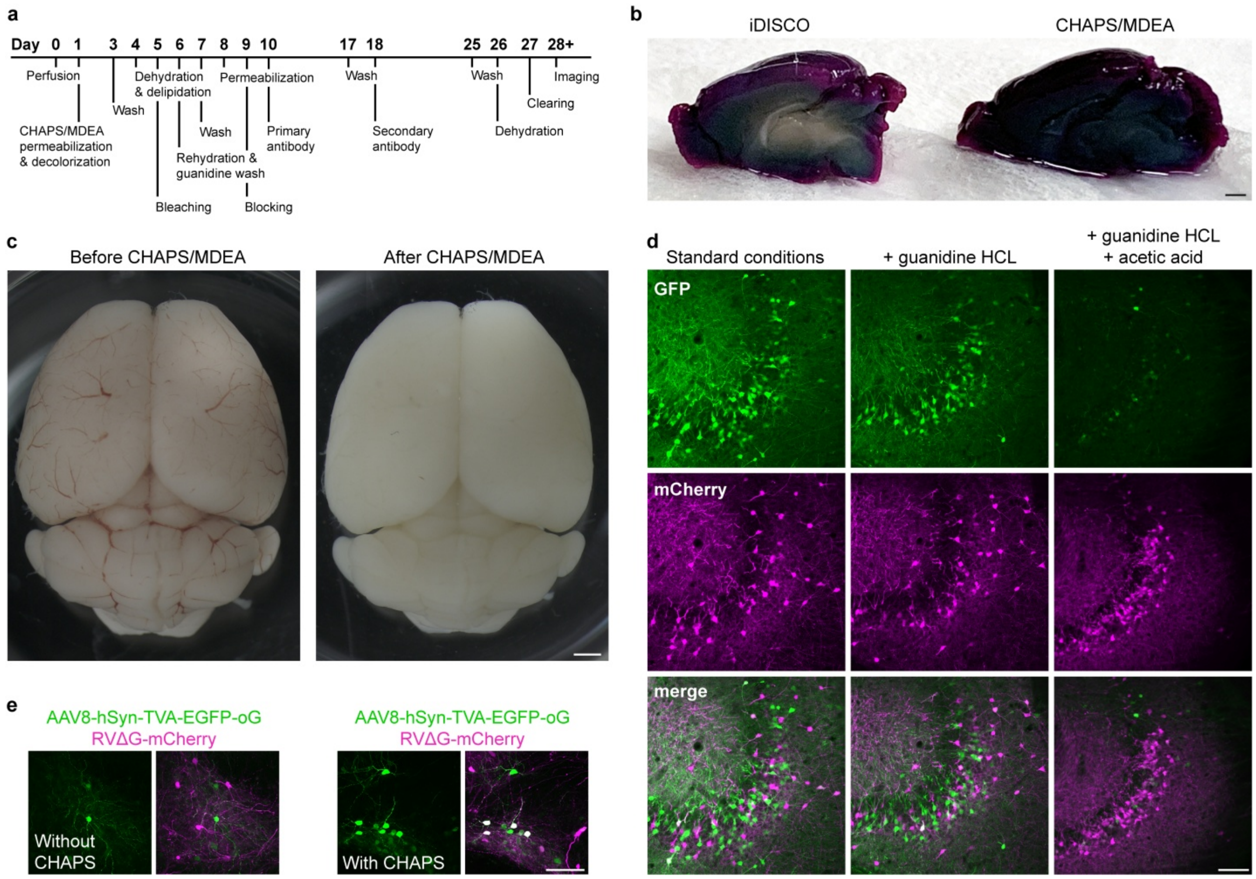

Supplementary Figure 3: Optimization of enhanced iDISCO+ protocol. a. Timeline of brain clearing and immunolabeling. **b.** Whole mouse brains incubated with purple food coloring overnight after permeabilization with iDISCO+ buffer or CHAPS/MDEA buffer for 48 h. Representative animals from *n* = 2 mice per condition. **c.** Unperfused mouse brain drop-fixed in 4% PFA overnight (left) and the same brain after 48 h of decolorization in 10% CHAPS/25% MDEA in PBS (right). Representative animal from *n* = 24 mice. **d.** Coronal section of hippocampus labeled for AAV helper virus (green) and input neurons (magenta) using standard immunostaining conditions, immunostaining with 4M guanidine HCl treatment or with 4M guanidine HCl and acetic acid treatment. Representative animals from *n* = 2 mice per condition. **e.** Coronal section of hippocampus labeled for AAV helper virus (green) and input neurons (magenta) with standard antibody diluent (left) and diluent containing 0.25% CHAPS (right). Representative animals from *n* = 2 mice per condition. Scale bar, 1mm (b, c); 100 μm (d, e).

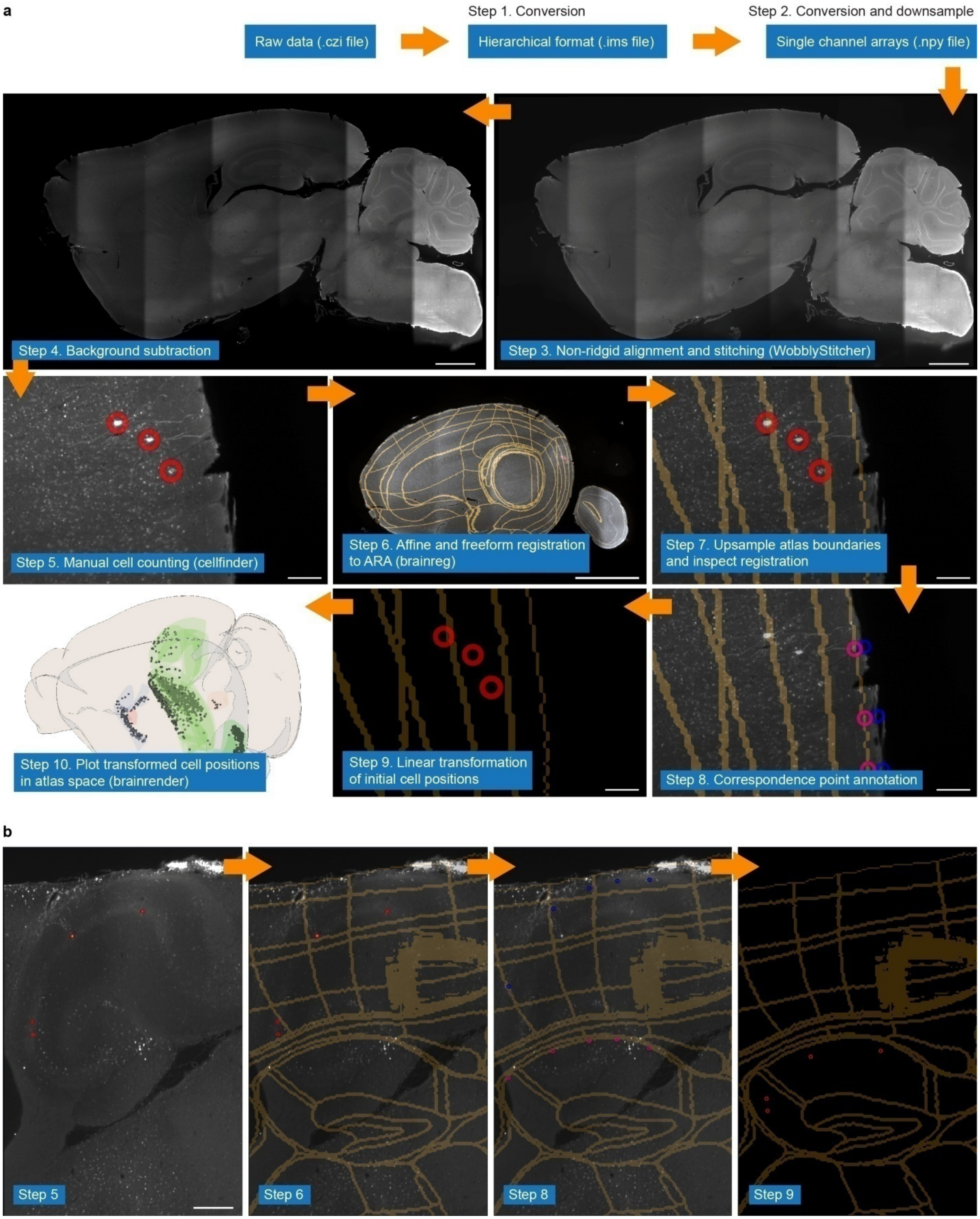
a
Step 1. Conversion
Step 2. Conversion and downsample
Raw data (.czi file)
Hierarchical format (.ims file)
Single channel arrays (.npy file)
Step 4. Background subtraction
Step 3. Non-ridgid alignment and stitching (WobblyStitcher)
Step 5. Manual cell counting (cellfinder)
Step 6. Affine and freeform registration to ARA (brainreg)
Step 7. Upsample atlas boundaries and inspect registration
Step 10. Plot transformed cell positions in atlas space (brainrender)
Step 9. Linear transformation of initial cell positions
Step 8. Correspondence point annotation
b
Step 5
Step 6
Step 8
Step 9

Supplementary Figure 4: Protocol for image registration and analysis. a. Raw data were initially converted from .czi to .ims file format. For each channel, data were downsampled by a factor of two in each dimension and converted to .npy format. 561 nm channel data was stitched using WobblyStitcher and exported as a stack of .tif files. For each animal, background intensity was measured and subtracted from all images in the stack. Individual cell positions were annotated using the graphical interface in cellfinder. 561 nm channel data were then down sampled to 10 μm isotropic resolution and registered to the Allen Reference Atlas. Atlas boundaries were upsampled to the original imaging resolution and overlaid over 561 nm channel data. For each image plane containing cells, atlas boundaries were manually inspected for accuracy. Registration error was corrected by annotating pairs of points corresponding to region boundaries (blue) and the respective atlas location to correct (magenta) to calculate transformation vector length. Representative animal from *n* = 9 uninjured mice. **b.** Atlas registration at the injury epicenter in a brain injured animal. Representative animal from *n* = 15 uninjured mice. Scale bars, 1mm (a, Steps 3, 4 and 6), 100 μm (a, Steps 5, 7-9) and 250 μm (b).

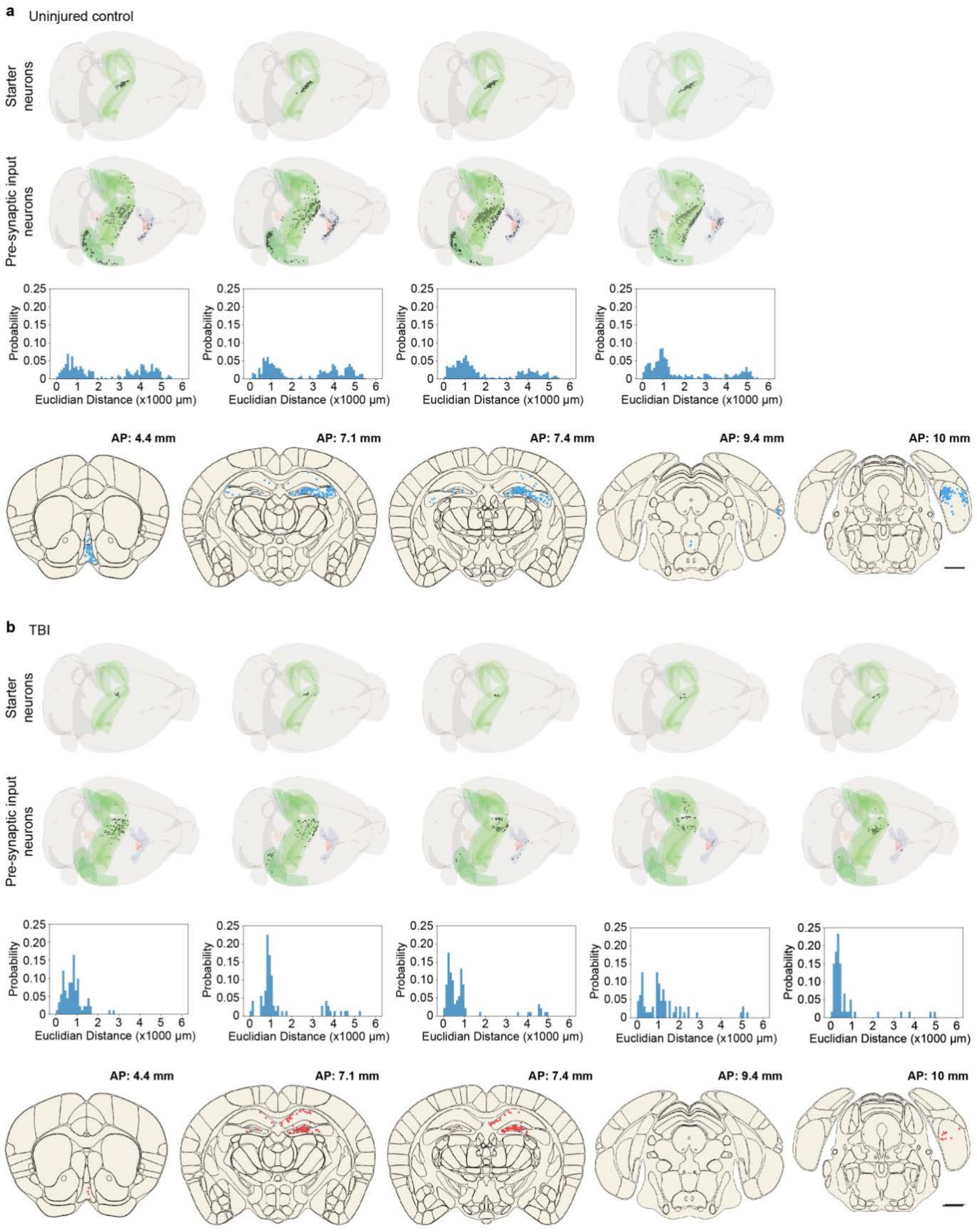

Supplementary Figure 5: Individual animal plots of input to hilar SST interneurons. a, b. Cell counts for each uninjured control (a) and brain injured animal (b). Top: whole-brain plots of starter cells and pre-synaptic input neurons annotated in standardized atlas space. Middle: histograms showing Euclidian distances at 100 µm bins. Black dots represent individual cells

registered in standardized atlas space. Shading indicates hippocampal formation (green), pallial regions (blue) and hypothalamus (red). Bottom: Schematic coronal sections (250 μm) showing individual rabies-labeled cells registered in standardized atlas space. Scale bar, 1 mm. Source data are provided as a Source Data file.

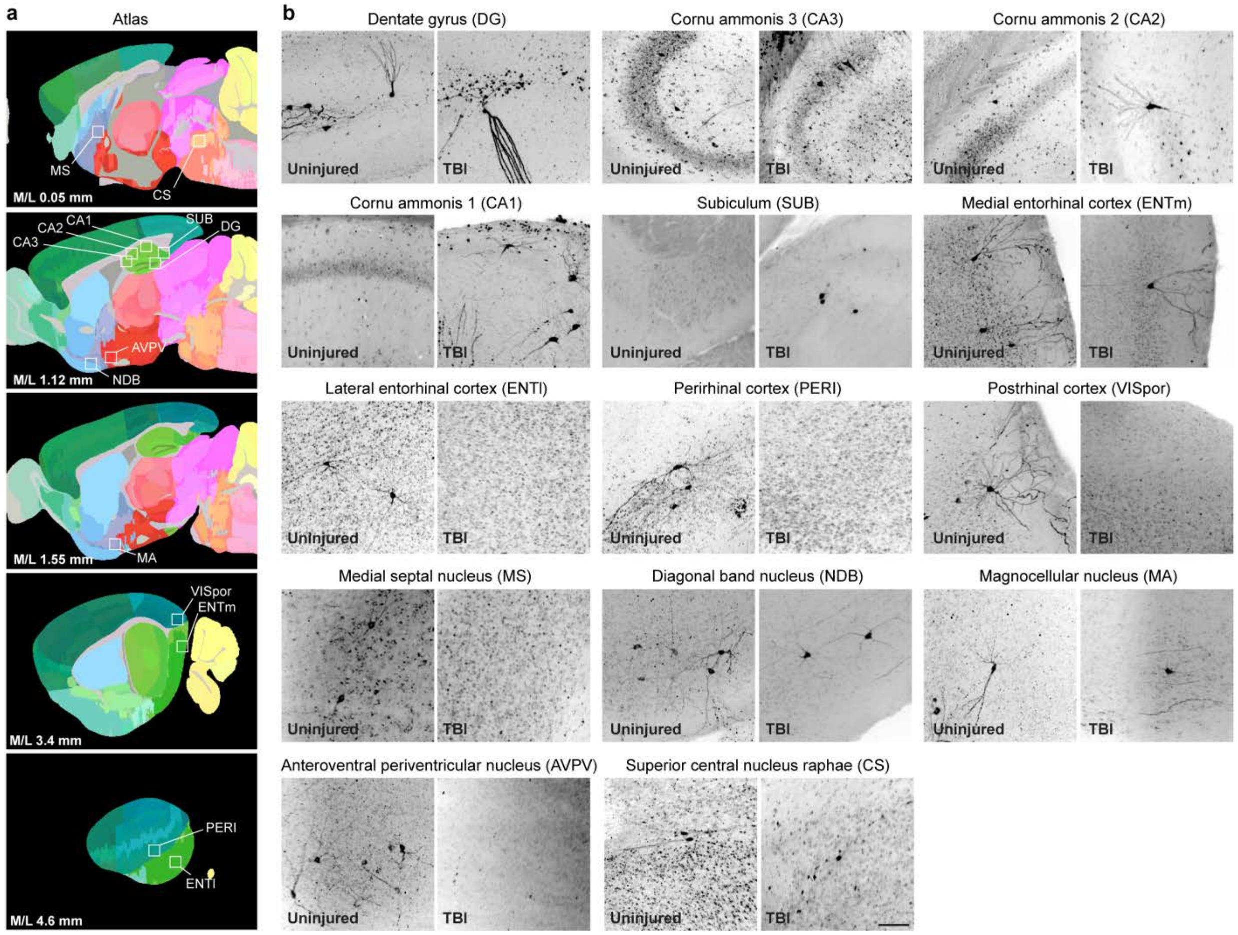

Supplementary Figure 6: Input neurons innervating hilar SST interneurons in hippocampus. a. Sagittal sections obtained from the ARA indicating brain regions where rabies-labeled cells were found. **b.** Maximum intensity projections (100µm) of neurons in whole brains providing direct input to hilar SST interneurons in the intact and damaged brain. Representative animals from *n* = 4 uninjured and 5 TBI mice. Scale bar, 100 µm.

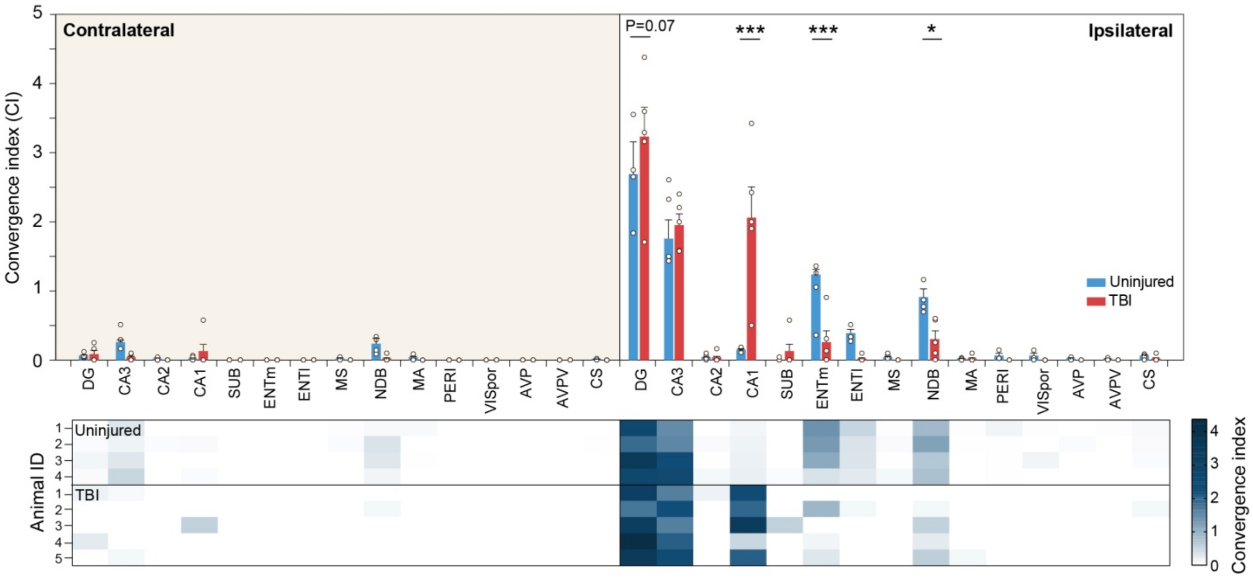

Supplementary Figure 7: Retrograde circuit tracing data represented as a convergence index. The number of rabies-labeled input neurons in each brain area were divided by the total number of starter cells counted for each animal to derive a convergence index for each brain area. Top: Average convergence index for each brain area. Bottom: individual values for each animal represented by a heat-map. ***P < 1.00E-15, CA1 (ipsilateral), ***P = 7.19E-04, ENTm (ipsilateral), *P = 3.29E-02, NDB (ipsilateral); two-way repeated-measures ANOVA with Bonferroni's post-hoc test; *n* = 4 uninjured and 5 TBI mice. Quantification and statistical analyses are provided in Supplementary Data 5. Error bars, s.e.m. A list of abbreviations is provided in Supplementary Data 2. Source data are provided as a Source Data file.

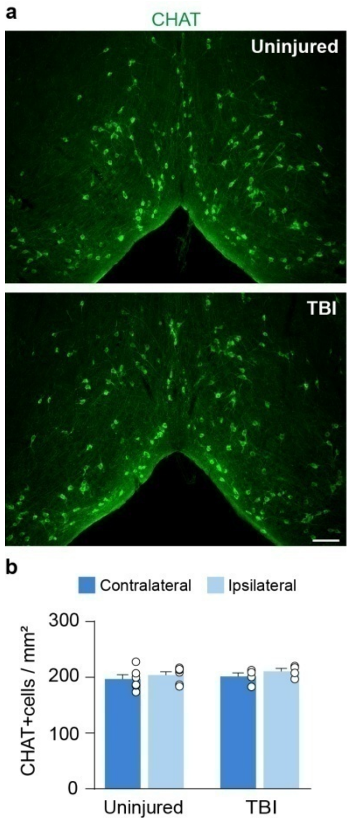

Supplementary Figure 8: Replication of CHAT immunostaining with conventional immunostaining in a separate cohort of animals. a. Coronal sections of NDB labeled for CHAT in an uninjured control and a brain injured animal. Scale bar, 100 µm. Representative animals from *n* = 6 uninjured and 4 TBI mice. **b.** Quantification of CHAT+ cell density. Error bars, s.e.m. Statistical analyses are provided in Supplementary Data 1. Source data are provided as a Source Data file.

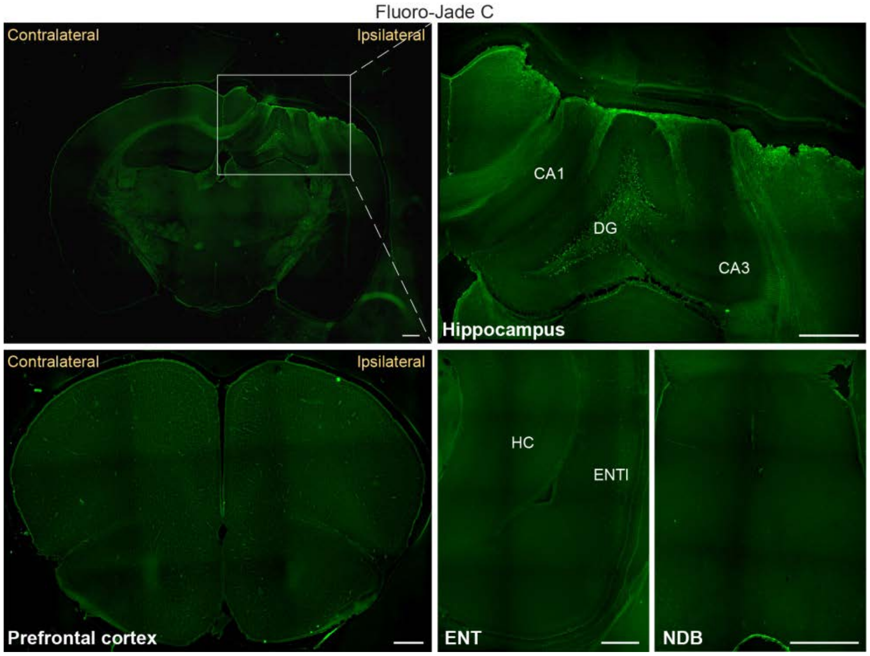

Supplementary Figure 9: TBI produces highly focal neurodegeneration. Coronal sections through dorsal hippocampus (HC), prefrontal cortex, entorhinal cortex (ENT) and diagonal band nucleus (NDB) labeled for fluoro-jade C 24 h after TBI. Representative animal from *n* = 3 TBI mice. Scale bar, 500 μm.

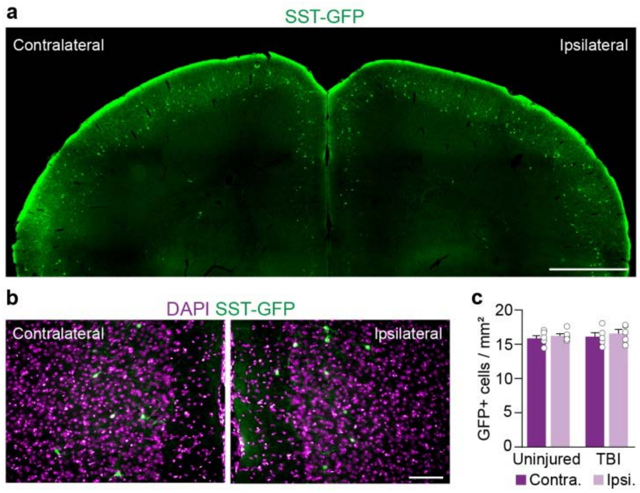

Supplementary Figure 10: SST interneurons are not reduced in PFC after TBI. a, b. Coronal section 8 wks after TBI labeled for SST-GFP (green) and DAPI (magenta). Representative animal from *n* = 5 TBI mice. **c.** Quantification of SST-GFP interneurons in uninjured and brain injured animals. *n* = 6 uninjured controls and 5 TBI mice. Statistical analyses are provided in Supplementary Data 1. Error bars, s.e.m.; scale bars, 1 mm (a), 100 μm (b). Source data are provided as a Source Data file.

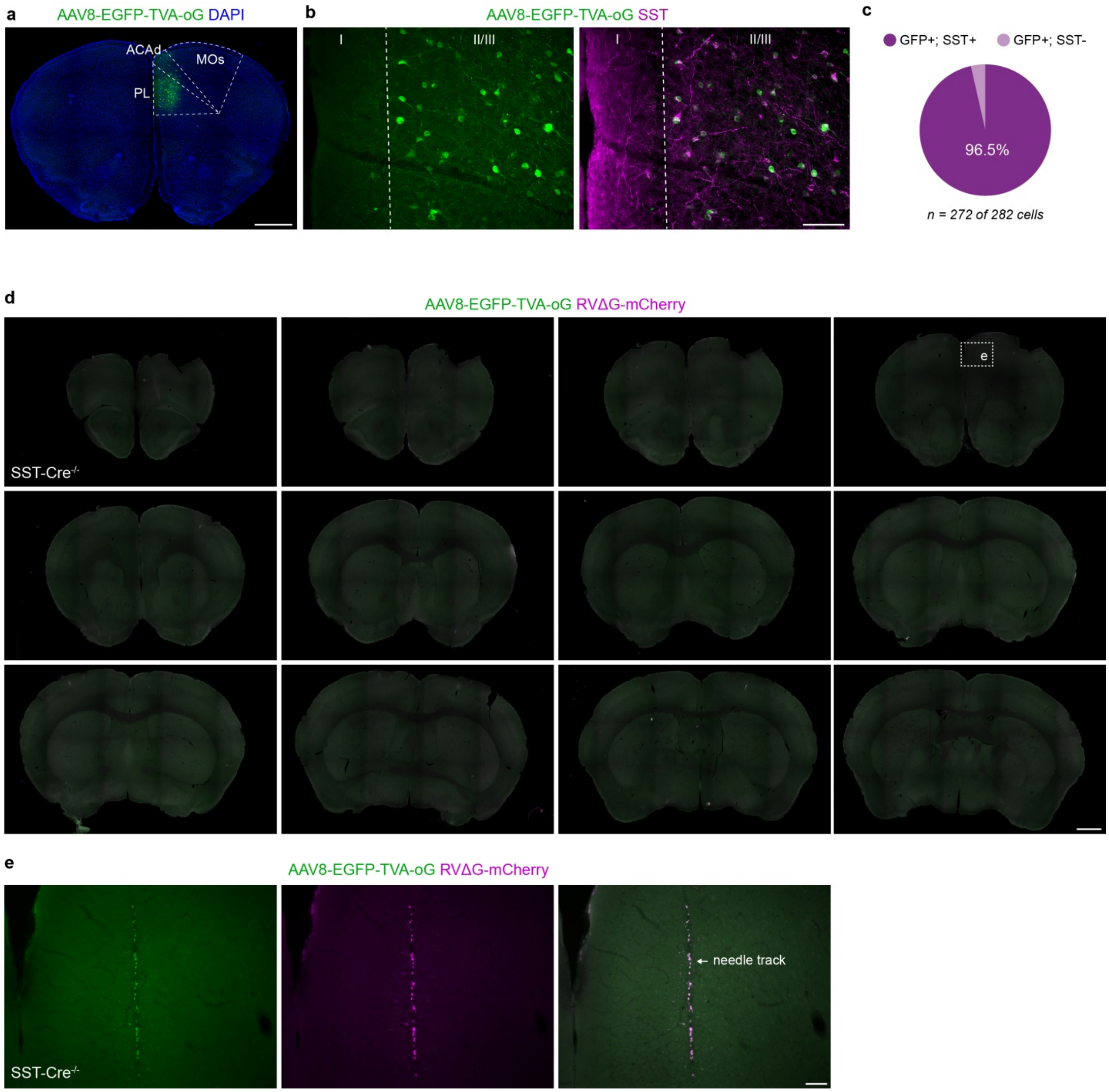

Supplementary Figure 11: Virus specificity in PFC. a, b. Coronal section of a control animal labeled for AAV-GFP helper virus (green), SST (magenta) or DAPI (blue) 3 wks after AAV injection into PFC. **c.** Proportion of GFP+ cells that express SST. **d.** Serial coronal sections every 300 μm through the entire brain of an adult SST-Cre-negative mouse labeled for AAV helper virus (green) and rabies (magenta). **e.** High magnification image of the injection site in PFC of the same SST-Cre-negative mouse shown in d. Scale bars, 1 mm (a, d), 100 μm (b, e).

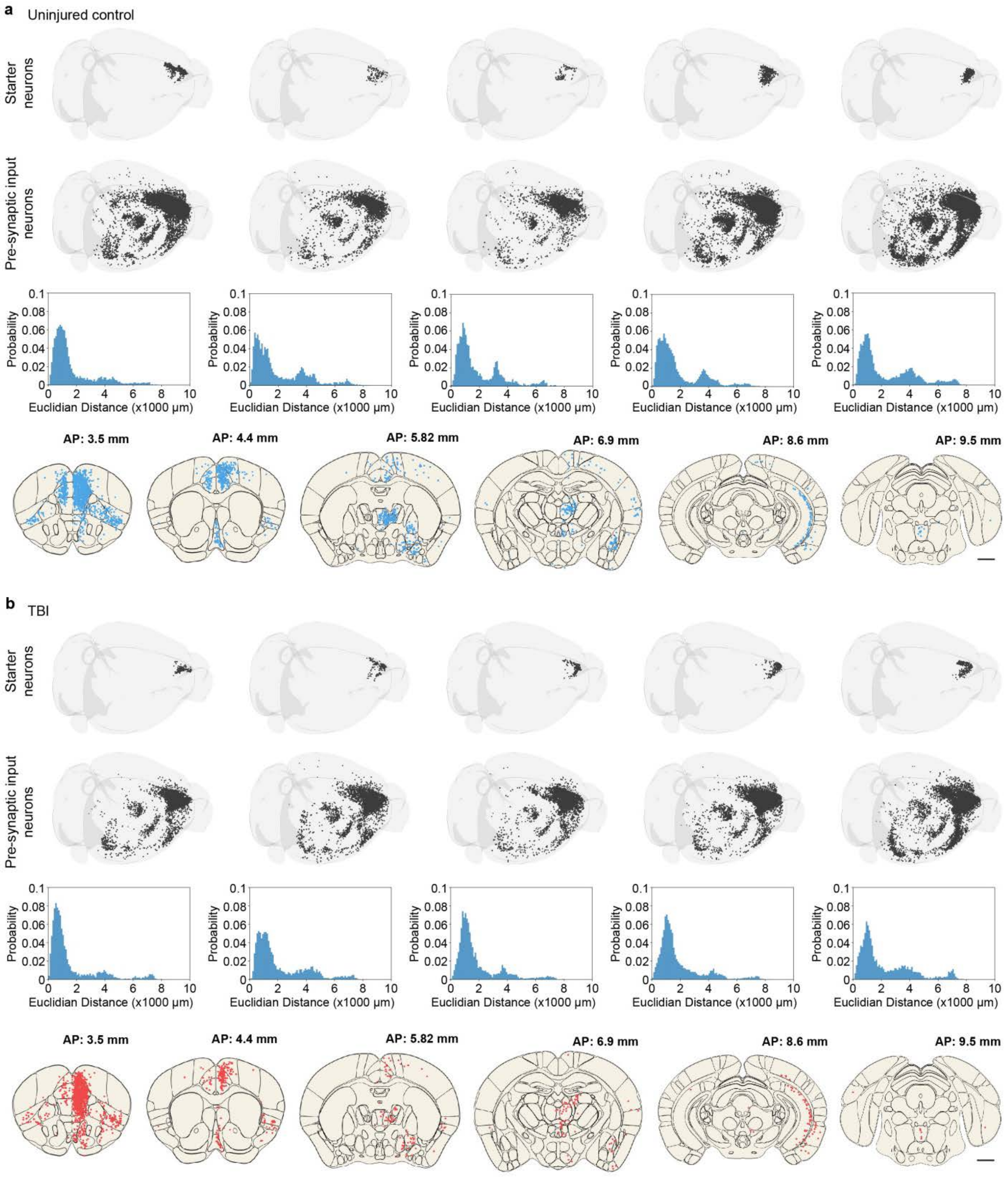

Supplementary Figure 12: Individual animal plots of input to PFC SST interneurons. a, b. Cell counts for each uninjured control (a) and brain injured animal (b). Top: whole-brain plots of starter cells and pre-synaptic input neurons annotated in standardized atlas space. Middle: histograms showing Euclidian distances at 100 µm bins. Black dots represent individual cells registered in standardized atlas space. Bottom: Schematic coronal sections (250 µm) showing

individual rabies-labeled cells registered in standardized atlas space. Scale bar, 1 mm. Source data are provided as a Source Data file.

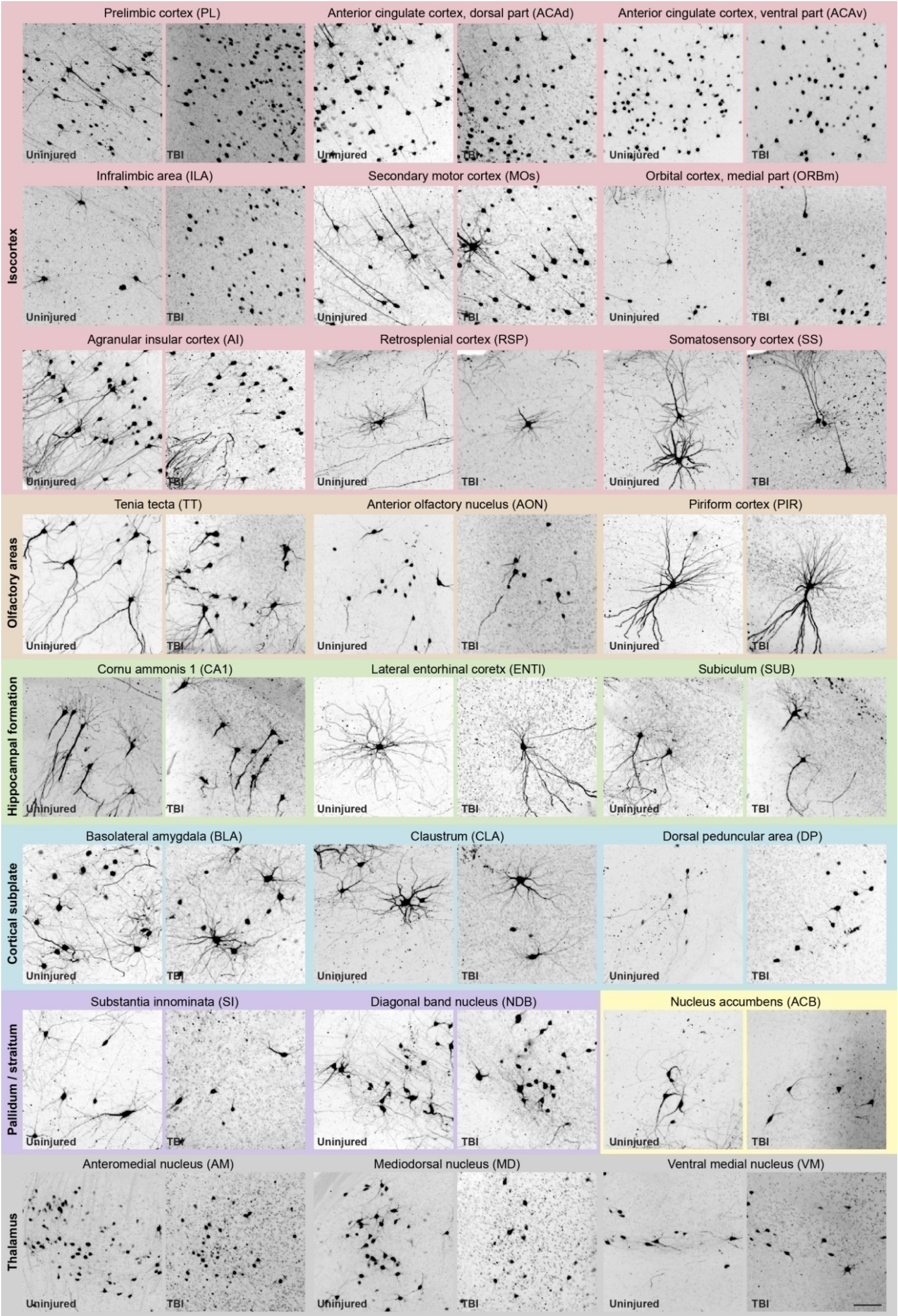

Isocortex
Prelimbic cortex (PL)
Anterior cingulate cortex, dorsal part (ACAd)
Anterior cingulate cortex, ventral part (ACAv)
Infralimbic area (ILA)
Secondary motor cortex (MOs)
Orbital cortex, medial part (ORBm)
Agranular insular cortex (AI)
Retrosplenial cortex (RSP)
Somatosensory cortex (SS)
Olfactory areas
Tenia tecta (TT)
Anterior olfactory nucleus (AON)
Piriform cortex (PIR)
Hippocampal formation
Cornu ammonis 1 (CA1)
Lateral entorhinal coretx (ENTl)
Subiculum (SUB)
Cortical subplate
Basolateral amygdala (BLA)
Claustrum (CLA)
Dorsal peduncular area (DP)
Pallidum / straitum
Substantia innominata (SI)
Diagonal band nucleus (NDB)
Nucleus accumbens (ACB)
Thalamus
Anteromedial nucleus (AM)
Mediodorsal nucleus (MD)
Ventral medial nucleus (VM)
Uninjured
TBI

Supplementary Figure 13: Input neurons innervating PFC SST interneurons. Maximum intensity projections (100 μm) of neurons providing direct input to PFC SST interneurons in the intact and damaged brain. Representative animal from *n* = 5 animals per group. Scale bar, 100 μm.

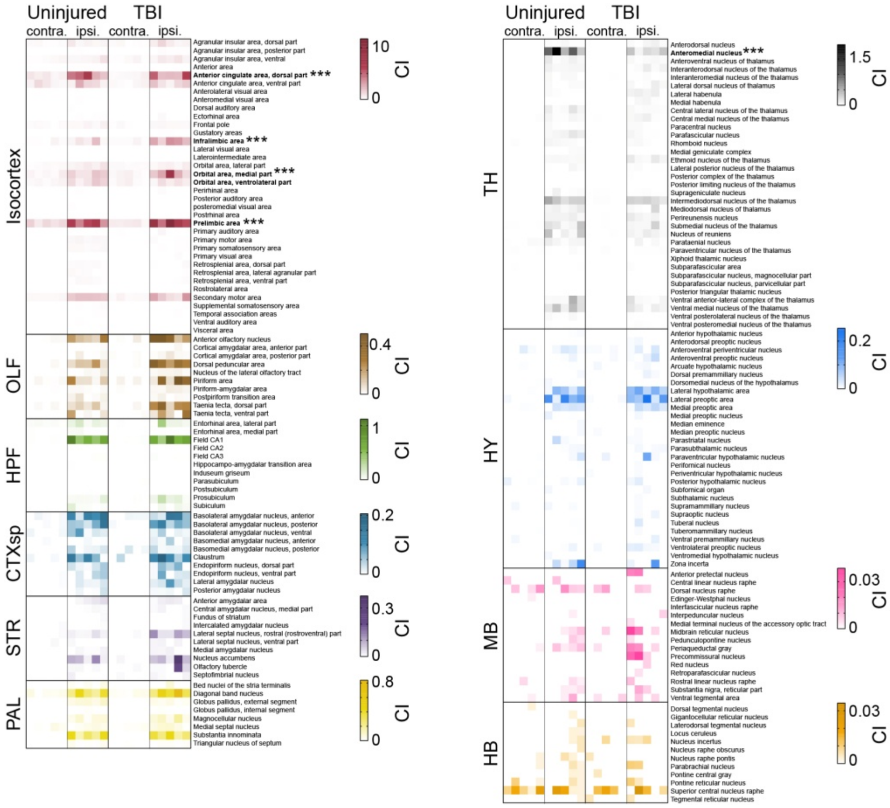

Supplementary Figure 14: Retrograde circuit tracing data in PFC represented as a convergence index. Heatmap showing convergence index for all 178 discrete brain areas providing input to SST interneurons in PFC. ***P = 4.17E-4, uninjured versus TBI (ACAd, ipsilateral), ***P< 1.00E-15, uninjured versus TBI (ILA, ipsilateral), ***P < 1.00E-15, uninjured versus TBI (ORBm, ipsilateral), ***P <1.00E-15, uninjured versus TBI (PL, ipsilateral),***P = 1.73E-04, uninjured versus TBI (AM, ipsilateral). Each box represents one animal. Statistical analyses are provided in Supplementary Data 9. A list of abbreviations is provided in Supplementary Data 2. Source data are provided as a Source Data file.

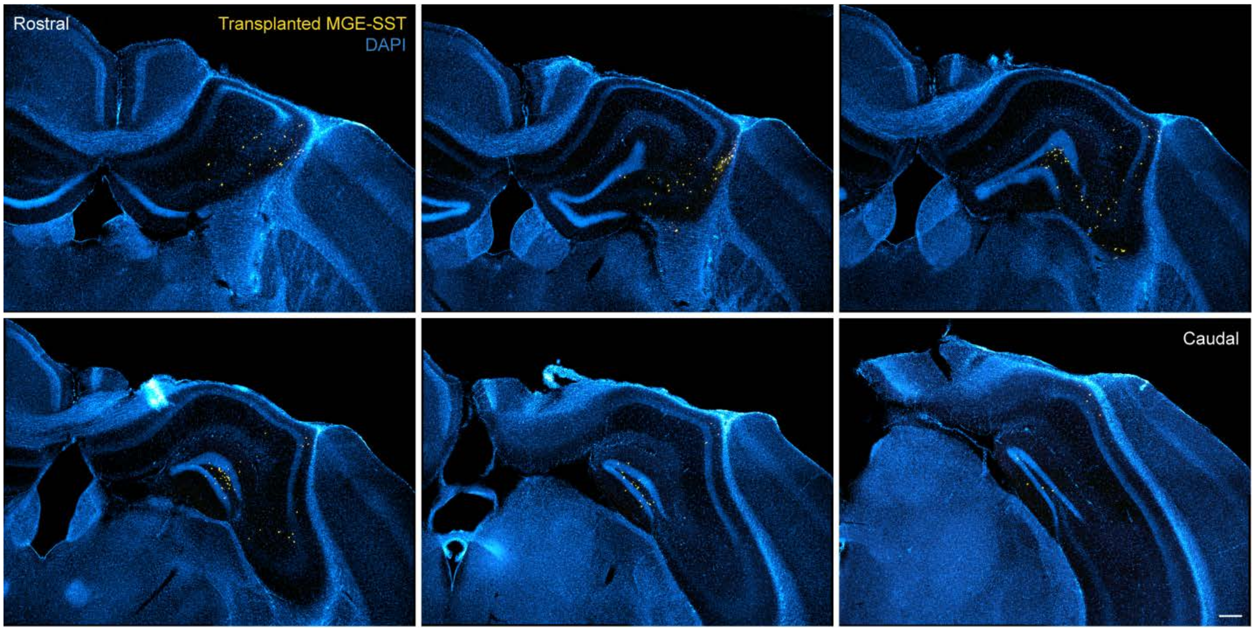

Supplementary Figure 15: Distribution of transplanted MGE-SST cells. Coronal sections spaced 300 µm apart showing the distribution of SST::Ai6-expressing interneurons 35 DAT into a mouse with TBI. Representative animal from *n* = 3 mice. Scale bar, 250 µm.

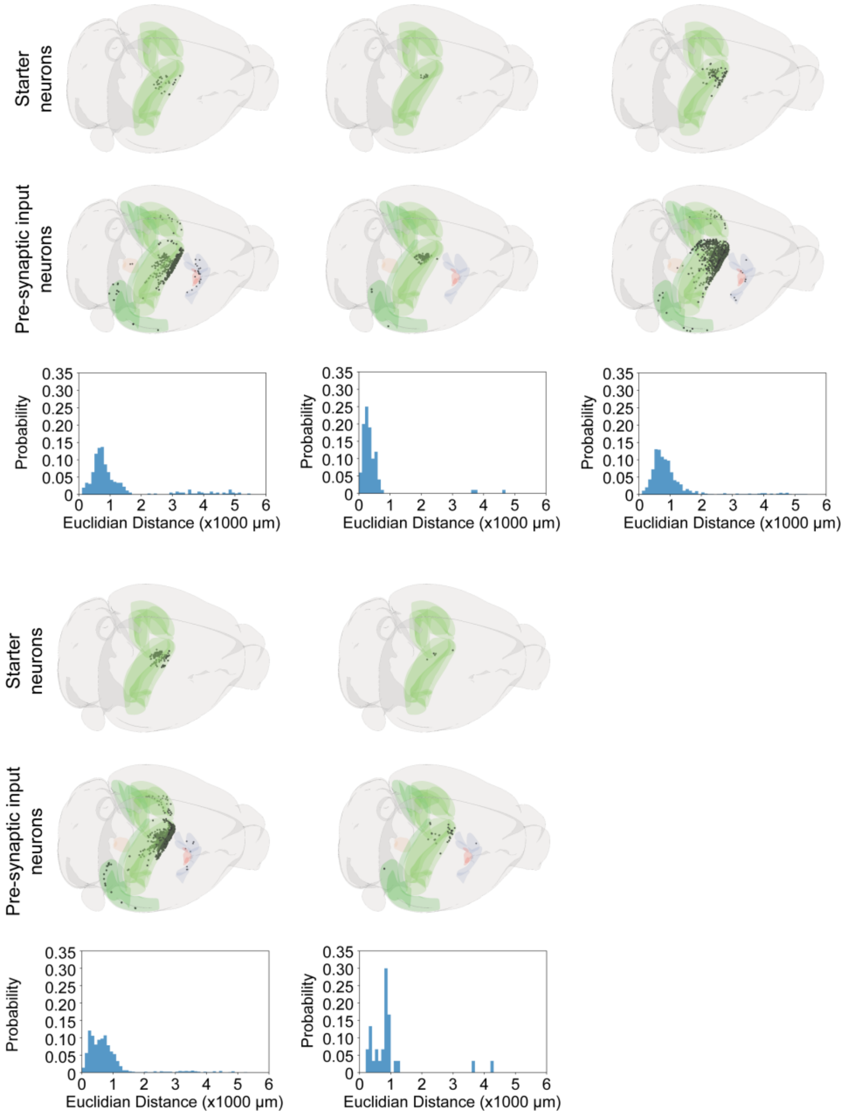

Supplementary Figure 16: Individual animal plots of input to transplanted SST interneurons. Top: whole-brain plots of starter cells and pre-synaptic input neurons annotated in standardized atlas space. Bottom: histograms showing Euclidian distances at 100 µm bins. Black dots represent individual cells registered in standardized atlas space. Shading indicates hippocampal formation (green), pallial regions (blue) and hypothalamus (red). Source data are provided as a Source Data file.

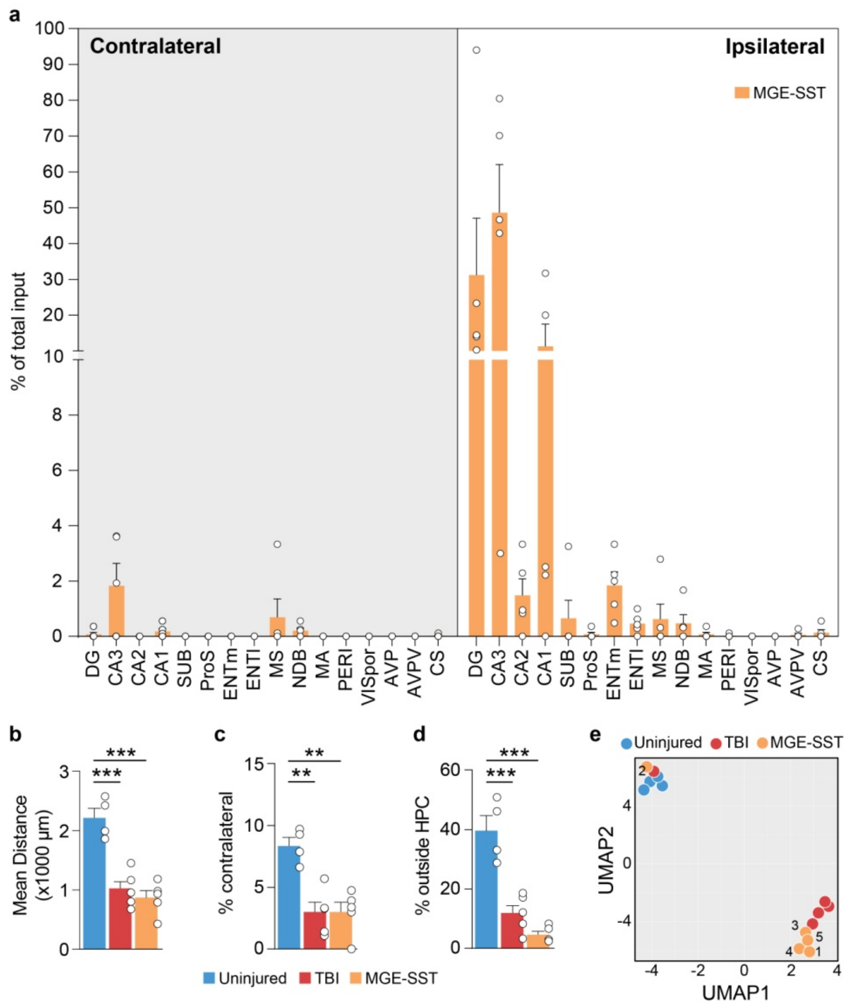

Supplementary Figure 17: Input to transplanted interneurons. a. Proportion of input neurons to transplanted SST interneurons found in each discrete brain area. *n* = 4 MGE-SST mice. **b.** Quantification of average Euclidian distance between transplanted starter cell centroid and input neuron positions. Uninjured: 2214.1 ± 169.9 μm, TBI: 1012.4 ± 136.7 μm, MGE-SST: 865.0 ± 125.9 μm; ****P* = 3.07E-04, uninjured versus TBI; ****P* = 1.13E-04, uninjured versus MGE-SST; P = 7.35E-01, TBI versus MGE-SST; one-way ANOVA with Tukey's post-hoc test, *n* = 4 uninjured, 5 TBI and 5 MGE-SST mice. **c.** Proportion of input neurons found in contralateral hemisphere. Uninjured: 8.4 ± 0.7 %, TBI: 3.0 ± 0.8 %, MGE-SST: 3.0 ± 0.8 %; ***P* = 1.83E-03, uninjured versus TBI; ***P* = 1.91E-03, uninjured versus MGE-SST, P= 9.99E-01, TBI versus MGE-SST; one-way ANOVA with Tukey's post-hoc test, *n* = 4 uninjured, 5 TBI and 5 MGE-SST mice. **d.** Proportion of input neurons found outside hippocampus. Uninjured: 39.6 ± 5.2 %, TBI: 11.8 ± 2.8 %, MGE-SST: 4.6 ± 1.1%; ****P* = 2.0E-04, uninjured versus TBI; ****P* = 2.45E-05, uninjured versus MGE-SST; P = 0.25, TBI versus MGE-SST; one-way ANOVA with Tukey's post-hoc test, *n* = 4 uninjured, 5 TBI and 5 MGE-SST mice. **e.** Uniform Manifold Approximation

and Projection (UMAP) clustering by % input neurons. Error bars, s.e.m. Source data are provided as a Source Data file.
