# Supplementary material for: Brain-wide reconstruction of inhibitory circuits after traumatic brain injury
Source: Nat Commun. 2022 Jun 14;13:3417. doi: 10.1038/s41467-022-31072-2 (PMC9197933; doi:10.1038/s41467-022-31072-2)
Supplement: Supplementary file 2 — Description to Additional Supplementary Information [file 41467_2022_31072_MOESM2_ESM.pdf]

Description of Additional Supplementary Files

Supplementary Movie 1: Input neurons to hilar SST interneurons in an uninjured mouse brain.

Supplementary Movie 2: Input neurons to hilar SST interneurons in an injured mouse brain.

Supplementary Movie 3: Input neurons to PFC SST interneurons in an uninjured mouse brain.

Supplementary Movie 4: Input neurons to PFC SST interneurons in an injured mouse brain.

Supplementary Movie 5: Input neurons to transplanted SST interneurons in an injured mouse brain.

Supplementary Data 1: Quantification of immunostaining experiments.

Supplementary Data 2: ARA nomenclature.

Supplementary Data 3: AP distribution of input to hilar SST interneurons.

Supplementary Data 4: Proportion of input to hilar SST interneurons.

Supplementary Data 5: Convergence index (CI) of input to hilar SST interneurons.

Supplementary Data 6: DV distribution of starter cells in PFC.

Supplementary Data 7: AP and ML distribution of input to PFC SST interneurons.

Supplementary Data 8: Proportion of input to PFC SST interneurons.

Supplementary Data 9: Convergence index (CI) of input to PFC SST interneurons.
